# Reliability of mobility measures in older medical patients with cognitive impairment

**DOI:** 10.1186/s12877-019-1036-z

**Published:** 2019-01-23

**Authors:** Tobias Braun, Christian Thiel, Ralf-Joachim Schulz, Christian Grüneberg

**Affiliations:** 1 Department of Applied Health Sciences, Division of Physiotherapy, Hochschule für Gesundheit (University of Applied Sciences), Gesundheitscampus 6-8, 44801 Bochum, Germany; 2 Faculty of Sports Science, Training and Exercise Science, Ruhr-University Bochum, Bochum, Germany; 3 Department of Geriatric Medicine, St. Marien-Hospital, Kunibertskloster 11-13, 50668 Cologne, Germany

**Keywords:** Older people, Mobility limitation, Dementia, Cognitive impairment, Outcome measure, Reliability, Measurement error, Minimal detectable change, Limits of agreement

## Abstract

**Background:**

Mobility is a key indicator of physical functioning in older people, but there is limited evidence of the reliability of mobility measures in older people with cognitive impairment. This study aimed to examine the test-retest reliability and measurement error of common measurement instruments of mobility and physical functioning in older patients with dementia, delirium or other cognitive impairment.

**Methods:**

A cross-sectional study was performed in a geriatric hospital. Older acute medical patients with cognitive impairment, indicated by a Mini-Mental State Examination (MMSE) score of ≤24 points, were assessed twice within 1 day by a trained physiotherapist.

The following instruments were applied: de Morton Mobility Index, Hierarchical Assessment of Balance and Mobility, Performance-Oriented Mobility Assessment, Short Physical Performance Battery, 4-m gait speed, 5-times chair rise test, 2-min walk test, timed up and go test, Barthel Index mobility subscale and Functional Ambulation Categories.

As appropriate, the intraclass correlation coefficient (ICC), Cohen’s kappa, standard error of measurement, limits of agreement and minimal detectable change (MDC) values were estimated.

**Results:**

Sixty-five older acute medical patients with cognitive impairment participated in the study (mean age: 82 ± 7 years; mean MMSE: 20 ± 4, range: 10 to 24 points). Some participants were physically or cognitively unable to perform the gait speed (46%), 2-min walk (46%), timed up and go (51%) and chair rise (75%) tests.

ICC and kappa values were above 0.9 in all instruments except for the gait speed (ICC = 0.86) and chair rise (ICC = 0.72) measures. Measurement error is reported for each instrument. The absolute limits of agreement ranged from 11% (de Morton Mobility Index and Hierarchical Assessment of Balance and Mobility) to 35% (chair rise test).

**Conclusions:**

The test-retest reliability is sufficient (> 0.7) for group-comparisons in all examined instruments. Most mobility measurements have limited use for individual monitoring of mobility over time in older hospital patients with cognitive impairment because of the large measurement error (> 20% of scale width), even though relative reliability estimations seem sufficient (> 0.9) for this purpose.

**Trial registration:**

German Clinical Trials Register (DRKS00005591). Registered 2 February 2015.

**Electronic supplementary material:**

The online version of this article (10.1186/s12877-019-1036-z) contains supplementary material, which is available to authorized users.

## Background

Aside from providing life-supporting interventions, the goal of hospital care and rehabilitation for older people with critical illness is to improve or preserve their health, functional independence and quality of life. Mobility and physical functioning are crucial health-related outcomes, which have an impact on this goal. Mobility is defined in the World Health Organisation’s International Classification of Functioning, Disability and Health (ICF) as “moving by changing body position or location or by transferring from one place to another, by carrying, moving or manipulating objects, by walking, running or climbing, and by using various forms of transportation” [1]. Mobility is a key indicator of physical functioning in older people, and common measures of mobility, such as gait speed or the timed up and go test (TUG), are used to assess these outcomes.

To monitor alterations in mobility, clinicians depend on reliable measurement instruments to provide trustworthy test scores over time (change scores). To differentiate real change from measurement error, sound evidence on the extent of the latter must be available. Test-retest reliability (relative reliability) concerns the extent to which scores of patients who have not changed are the same for repeated measurement over time [2]. Classic measurement theory assumes that every measurement, or obtained score, consists of a true component and an error component, and all variability within a person’s score is viewed as measurement error [3]. Possible facets of variability in repeated test scores can be instrumented-based, rater-based or subject-based (biological variability) [3]. Thus, measurement error (absolute reliability) is the systematic and random error of a patient’s score that is not attributed to true changes in the construct to be measured [2]. Parameters of measurement error are the standard error of measurement, the limits of agreement proposed by Bland and Altman, and the minimal detectable change (MDC) [2, 4, 5]. The MDC is defined as a change beyond measurement error; it “represents the spread of the distribution of change scores that would be expected if no true change had occurred” [4].

A significant proportion of older hospital patients presents with cognitive impairment, which typically results from chronic conditions (e.g. dementia) or temporal syndromes (e.g. delirium). The in-hospital prevalence for dementia is estimated to be between 13 and 63% [6]. Approximately 20 to 27% of older acute patients present with delirium [7, 8]. Obtaining reliable performance-based test scores from older people with dementia can be challenging [9–11]. Proposed requirements include the ability to comprehend test commands, the ability to develop an adequate motor action and sequence, the ability to recollect both during execution, as well as the patient’s adequate motivation and attention during testing [9]. Especially in acute medical patients with dementia, delirium or other cognitive impairment, these requirements may vary over time and influence the within-subject variance of the test performance.

Limited information exists on the reliability of mobility measures in older people with dementia, delirium or other cognitive impairment [12, 13]. The methodological quality of the few, mostly small-scale studies varies, and for the most commonly used instruments, there is conflicting evidence on test-retest reliability. By way of example, for the TUG, intraclass correlation coefficients (ICCs) between 0.56 and 0.96 have been reported [11, 14–17]. Test-retest reliability estimates also vary considerably for gait speed measures (ICC = 0.57 to 0.97) [16–19], and timed chair rise tests (ICC = 0.80 to 0.97) [15, 17, 20–22]. Reliability estimates of such single-component mobility instruments are based on studies performed with older community-dwelling (outpatient) people or nursing-home residents with cognitive impairment.

For multicomponent instruments, which are considered more construct valid and applicable in the hospital setting [23, 24], such as the Hierarchical Assessment of Balance and Mobility (HABAM) [25], the Short Physical Performance Battery (SPPB) [26], Tinetti’s Performance Oriented Mobility Assessment (POMA) and the de Morton Mobility Index (DEMMI) [27], evidence on test-retest reliability in older hospital patients with cognitive impairment has not yet been established.

We have recently examined the psychometric properties of the DEMMI in older individuals with dementia, delirium or other cognitive impairment, providing first evidence for the DEMMI to be a feasible, unidimensional and construct valid measurement instrument of mobility in this population [28]. Since we have not analysed reliability in this study, the main objective of the present study was to examine the test-retest reliability of the DEMMI. Given the lack of evidence on the reliability of mobility measures in older people with cognitive impairment, the secondary objective was to examine the test-retest reliability of several other commonly used measures of mobility in older acute medical patients with dementia, delirium or other cognitive impairment based on the available data set.

## Methods

### Design and setting

This cross-sectional study is a sub-analysis of the reliability data generated in a primary study on the psychometric properties of the DEMMI in a consecutive sample of older acute medical patients with cognitive impairment [28]. The primary study was approved by the Ethical Review Board of the University of Cologne (registration number 2014–05), conducted according to the ethical principles of the Declaration of Helsinki (2013), a priori registered in the German Clinical Trials Register (DRKS00005591) and performed in a geriatric hospital in Cologne, Germany. All participants provided ongoing, written informed consent. Additional guardian informed consent was obtained for every participant with a legal representative and for every participant considered to have limited capability to understand the study procedures. The latter was determined by a consortium composed of the ward physician, the primary nurse and the relatives, if appropriate. Proposed recommendations of the STrengthening the Reporting of Observational studies in Epidemiology (STROBE) statement for cross-sectional studies as well as the Guidelines for Reporting Reliability and Agreement Studies (GRRAS) were followed [29, 30].

Participants with cognitive impairment included in the primary study were assessed with a comprehensive set of mobility measures immediately after hospital admission (baseline sample). To assess test-retest reliability, all baseline mobility measures were repeated in a sub-sample of the baseline sample participants. The present study reports the test-retest reliability and measurement error of commonly used measurement instruments of mobility and physical functioning, including the corresponding subscales.

### Participants

Participant enrolment was from 4 February to 11 December 2015. We defined 91 screening days, which were unsystematically spread across the study period. All acute geriatric inpatients consecutively admitted to the clinic on one of the screening days were screened for eligibility. A sample of 153 patients was included and constituted the baseline sample of the primary study [28].

Patients were eligible if they were admitted to one of the acute geriatric wards of the hospital, ≥60 years old and presented with cognitive impairment as indicated by a Mini-Mental State Examination (MMSE) score of ≤24 points [31]. Exclusion criteria were: documented contraindications for mobilisation, physician-directed partial weight-bearing of the lower extremity, isolation for infection, impending death, coma or severely impaired vigilance, acute major organ failure, blindness, deafness, severe dysphasia, German language barrier, or any acute psychiatric or medical/physical condition whereby mobility measurements could lead to a worsening of the health state.

### Procedures

Eligible participants were examined within 7 days after hospital admission by the primary investigator (TB), a physiotherapist with 7 years of clinical and academic working experience who was well trained in the administration of the measurement instruments (has used each instrument in more than 200 cases prior to this study). In a single session, a comprehensive set of commonly used performance-based measurement instruments of mobility was administered in a standardised order, starting with the least physically challenging tests. Similar items in different assessments were only performed once to reduce participant’s burden, e.g. standing with both feet together is required in the DEMMI and the Performance Oriented Mobility Assessment (POMA). In a sub-sample of eligible participants, all measures were repeated by the same assessor on the same day and in the same environment. The single independent rater was well informed of each participant’s medical condition, such as diagnoses and level of cognitive impairment. The rater was not informed of the mobility capacity of each participant in detail (e.g. blinded towards routine physiotherapy outcome scores and walking aid use). In the retest session, the rater was not blinded towards the results of the first session.

A reliability analysis should be based on scores of patients whose medical condition has not changed (stable/unchanged) [2]. In the present study, the baseline assessment session was usually performed in the morning, and the retest was usually done in the afternoon. Both assessment sessions were always administered on Saturdays. On weekdays, throughout the day, participants took part in a number of medical treatments and other interventions as part of usual care, making a change in the participants’ physical and mental condition very likely (e.g. fatigue, pain, exhaustion or motor learning). On Saturdays, usual care therapy interventions were only applied to a small number of severely affected individuals in the study hospital. To explicitly include stable/unchanged participants, according to the definition of reliability [2], the intra-day retest assessment was only performed on participants assessed on Saturdays who did not participate in any diagnostic procedures or rehabilitation sessions (e.g. physical or occupational therapy) in between study assessments. Participants who reported any change in their physical or mental condition with respect to the first assessment (e.g. fatigue, pain or dizziness) were considered unstable and excluded. The nursing staff and the medical charts were consulted to validate the participants’ perception of stability.

Socio-demographic data were taken from the medical records and from hospital administrative data. The MMSE [31], the Clock Drawing Test [32] and the 15-item short form of the Geriatric Depression Scale [33] were administered by the occupational therapy staff of the hospital as part of routine care. Diagnoses and medical symptoms that could be causal for the participants’ cognitive impairment were extracted from the final hospital discharge reports.

### Measurements

In this study, 10 performance-based measures of the mobility capacity of older people were applied in the following order: DEMMI [27, 34], HABAM [35, 36], POMA [37], TUG [38], SPPB [39], 4-m gait speed (as part of the SPPB), 5-times chair rise test (5xCRT; as part of the SPPB), 2-min walk test [40], Barthel Index mobility subscale [41], and Functional Ambulation Categories (FAC) [42]. Additional file 1 provides a detailed description of the assessment procedures and all measurement instruments and their subscales.

Table 1 presents a clustered overview of the measurement instruments examined in this study according to the ICF mobility domain components captured by each instrument. According to this evaluation, instruments are separated into single-component and multi-component measures, depending on the number of mobility domains included. The classification in Table 1 is the consensus of the authors, informed by the classifications reported by other authors [24, 43].

**Table 1:** Mobility domain components of each measurement instrument classified according to the ICF

Domain components		Multi-component measurement instruments						Single-component measurement instruments			
		DEMMI	HABAM	POMA	SPPB	TUG	BI mobility subscale	FAC	Gait speed	5xCRT	2minWT
Changing and maintaining body position (d410 – d429)											
d410	Changing basic body position	X	X	X	X	X	X			X	
d415	Maintaining a body position	X	X	X	X						
d420	Transferring oneself		X				X				
Carrying, moving and handling objects (d430 – d449)											
d430	Lifting and carrying objects										
d435	Moving objects with lower extremities										
d440	Fine hand use										
d445	Hand and arm use										
Walking and moving (d450 – d469)											
d450	Walking	X	X	X	X	X	X	X	X		X
d455	Moving around		X				X				
d460	Moving around in different locations										
d465	Moving around using equipment	X	X	X		X	X				
Moving around using transportation (d470 – d489)											
d470	Using transportation										
d475	Driving										
d480	Riding animals for transportation										

The domain components (constructs) of each instrument are classified according to the domain “mobility” (Activities and Participation, Chapter 4) described in the World Health Organization’s ICF. The ICF Mobility definition is: “Moving by changing body position or location or by transferring from one place to another, by carrying, moving or manipulating objects, by walking, running or climbing, and by using various forms of transportation”

*Abbreviations*: *ICF* International classification of functioning, disability and health (ICF), *DEMMI* De Morton mobility index, *HABAM* Hierarchical assessment of balance and mobility, *POMA* Performance oriented mobility assessment, *SPPB* Short physical performance battery, *TUG* Timed up and go test, *BI* Barthel index, *FAC* Functional ambulation categories, *5xCRT* 5 times chair rise test, *2minWT* 2-min walk test

### Statistical analysis

Data were analysed using SPSS 21.0 (IBM Corp., Armonk, New York) and Microsoft Excel 2016 (Microsoft Office, Redmond, Washington). Descriptive statistics were used to present sample characteristics. Statistical significance was set at *p* < 0.05.

### Reliability

#### Test-retest reliability

For all continuous outcomes, the relative intra-day test-retest reliability was examined using the intra-class correlation coefficient model 2.1 (two-way random effects model; ICC_AGREEMENT_) [4]. The ICC_AGREEMENT_ was calculated by dividing the systematic differences between the “true” scores of patients by the error variance, which consists of the systematic differences between the true scores of patients, the variance due to systematic differences between the two measurements, and the residual variance [4]. For the categorial outcome FAC, we determined the relative test-retest reliability using a weighted kappa with linear weights [4]. ICC and Ƙ ≥0.7 were deemed acceptable for group-comparisons, whereas ICC and Ƙ ≥0.9 were deemed acceptable for individual measurements over time [44, 45]. The test-retest reliability was additionally examined for sub-groups by gender.

For the retest sub-sample, the sample size was determined a priori and guided by the following three approximations: (1) For the main measure, the DEMMI, a minimum of 38 participants was needed based on the assumption of two occasions, a planning value of ICC = 0.92 reported by others [46] and the desired 95% confidence interval (CI) with a width of 0.10 [47]. (2) The COnsensus-based Standards for the selection of health Measurement INstruments (COSMIN) group recommends at least 30, 50 or 100 participants for a reliability study to have a “fair”, “good” or “excellent” sample size, respectively [2, 48]. (3) For measures of gait and sit-to-stand transfers, floor effects of approximately 50% were expected [11, 49–51]. To reach a “fair” sample size for such instruments subjected to large floor effects and missing values, we intended to re-assess at least 60 participants (*n* ≥ 30 after 50% drop-out).

#### Measurement error: standard error of measurement

For the continuous outcomes, the standard error of measurement (SEM_AGREEMENT_) was calculated using the same variance components used for the ICC_AGREEMENT_ calculation. The SEM_AGREEMENT_ was calculated using the square root of the variance between the two occasions and the error variance of the ICC_AGREEMENT_ [4].

For categorial measures, no parameters of measurement error can be calculated that quantify the measurement error in the units of measurement. To quantify agreement for the FAC, the percentage of measurements classified in the same FAC categories was calculated [52].

#### Measurement error: limits of agreement/Bland and Altman plot

The method of Bland and Altman was used to illustrate agreement between the baseline and retest measures of each instrument [5]. The 95% limits of agreement require homoscedasticity and normally distributed differences [53]. A positive Kendall’s tau (τ) correlation between the absolute differences and the corresponding means > 0.1 was deemed to denote heteroscedasticity [54]. In case of heteroscedastic data, the following formula was used to calculate the limits of agreement: $-2\mathrm{X}\ \frac{\left({10}^a-1\right)}{\left({10}^a+1\right)} and+2\mathrm{X}\ \frac{\left({10}^a-1\right)}{\left({10}^a+1\right)}$, where *a* = 95% limits of agreement of the 10 log transformed data, and X = the mean score [55]. We added bar charts for frequencies of differences to allow better interpretation.

#### Measurement error: minimal detectable change

The minimal detectable change (MDC) with 90 and 95% confidence, a quantification of absolute agreement, was calculated based on the test-retest reliability data as MDC_90_ = 1.64*√2*SEM_AGREEMENT_ and MDC_95_ = 1.96*√2*SEM_AGREEMENT_, respectively. The MDC_95_ (MDC_90_) is defined as the minimal amount of change that needs to occur between repeated assessments in an individual to exceed, with 95% (90%) confidence, the error of the measurement [56]. For all scales that consist of whole numbers only (DEMMI, HABAM, POMA, SPPB and Barthel Index mobility subscale), MDC values were rounded up to whole numbers.

## Results

**Fig. 1:**
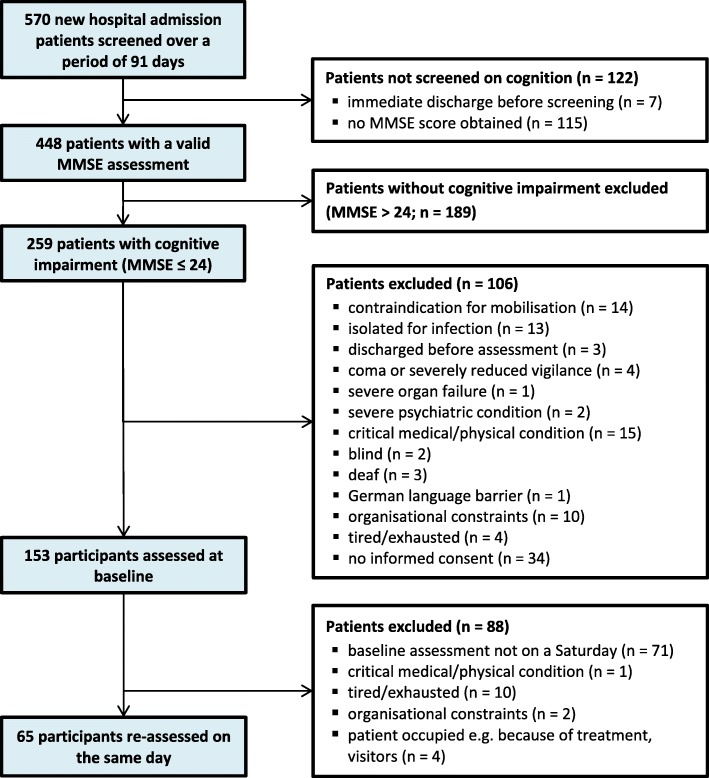
Flow chart of study participants (MMSE = Mini-Mental State Examination)

The baseline sample included 153 participants with cognitive impairment, of which 65 stable/unchanged participants were re-assessed within 1 day (participant flow: Fig. 1; admission characteristics: Table 2).

**Table 2:** Characteristics of participants (*n* = 65)

Characteristic	Value
Age, years	82 ± 7 (71–97)
Gender: male/female, n (%)	29/36 (45/55)
Pre-clinical living situation: home alone/home with family or relatives/ institutionalized, n (%)	34/29/2 (52/45/3)
Time between admission and assessment, days	2.9 ± 1.2 (1–5)
Total length of stay on the acute ward, days	19 ± 10 (7–64)
Primary diagnosis according to ICD-10 categories	
IX Circulatory, n (%)	11 (17)
X Respiratory, n (%)	5 (8)
XIII Musculoskeletal, n (%)	5 (8)
XVIII Symptoms, signs and abnormal clinical and laboratory findings, not elsewhere classified, n (%)	7 (11)
XIX Injury, poisoning and certain other consequences of external causes	24 (37)
Other, n (%)	13 (20)
Potential reasons for a cognitive impairment reported in the medical chart (diagnosis, symptom, medical sign; double-counts due to multi-morbidity)	
None reported, n (%)	21 (32)
Alzheimer’s dementia, n (%)	3 (5)
Vascular dementia, n (%)	6 (9)
Dementia, not specified, n (%)	15 (23)
Parkinson’s disease, n (%)	4 (6)
Stroke, n (%)	8 (12)
Depression, n (%)	15 (23)
Delir, n (%)	11 (17)
Other (psychosis, alcohol abuse), n (%)	3 (5)
In-hospital walking aid	
Wheeled-walker/rollator, n (%)	23 (35)
None, n (%)	7 (11)
Cane/single crutch, n (%)	7 (11)
Other, n (%)	2 (3)
Non-ambulatory (wheelchair), n (%)	26 (40)
Ambulation	
Independent walkers (FAC ≥ 4), n (%)	27 (42)
Not ambulatory or dependent walkers (FAC ≤ 3), n (%)	38 (58)
Barthel Index, 0–100 points	
Valid/missing, n (%)	64/1 (98/2)
Mean score, points (*n* = 148)	45 ± 24 (0–90)
Mini Mental State Examination, 0–30 points	
Severe cognitive impairment, 0–9 points, n (%)	0 (0)
Moderate cognitive impairment, 10–18 points, n (%)	19 (29)
Mild cognitive impairment, 19–24 points, n (%)	46 (71)
Mean score, points	20 ± 4 (10–24)
Median score, points	21 (18–23)
Clock Drawing Test, 1–6 points	
Unsuspicious: 1–2 points, n (%)	8 (12)
Suspicious: 3–6 points, n (%)	40 (62)
Missing/not possible, n (%)	17 (26)
Mean score, points (*n* = 122)	4.0 ± 1.3 (1–6)
Geriatric Depression Scale short form, 0–15 points	
Normal: 0–4 points, n (%)	30 (46)
Mildly depressive: 5–8 points, n (%)	17 (26)
Moderately depressive: 9–11 points, n (%)	8 (12)
Severely depressive: 12–15 points, n (%)	4 (6)
Missing/not possible, n (%)	1/5 (2/8)
Mean score, points (*n* = 59)	5 ± 3 (0–13)

Values are presented as mean ± standard deviation (range) or median (interquartile range)

*Abbreviations*: *ICD-10* International classification of diseases 10th version, *na* Not applicable, *FAC* Functional ambulation categories

Twenty-nine percent of participants presented with a moderate cognitive impairment, and 71% presented with a mild cognitive impairment. Thirty-seven percent of participants were diagnosed with dementia, while 17% were diagnosed with delirium. The mean time span between the cognitive assessment and the study assessments was 2.6 ± 1.2 (range: 0–5) days. Approximately one out of two participants (49%) reported a fall and its consequences as the main reason for their hospital admission.

Most participants (*n* = 38, 58%) were unable to walk or needed some kind of assistance for walking. Of the 65 participants, 30 (46%) were not physically able to perform the 2-min walk test and the gait speed measure over 4 m. These participants were generally either not able to walk at all, or they required assistance from one or more people to walk. The 5xCRT test could be evaluated in only 16 (25%) participants due to insufficient sit-to-stand transfer abilities, mainly based on limited lower limb strength, in the other 49 participants (75%). These 49 participants were not able to complete a sit to stand transfer without arms during the DEMMI administration (item #6). The TUG could not be assessed in 32 (49%) participants at baseline due to physical impairment (*n* = 31) and limited understanding of the test instructions (*n* = 1).

Reliability assessments were performed in the very early phase after hospital admission, with 3 days on average and within 5 days for every participant. The retest assessment was performed 218 ± 86 (range: 60–405) minutes after the first assessment. The time span between both assessments was ≤2 h for 11 participants (17%), between 2.25 and 4 h for 30 participants (46%), between 4.25 and 6 h for 22 participants (34%) and > 6 h for 2 participants (3%).

### Reliability

#### Test-retest reliability

Data on test-retest reliability are shown in Table 3 and in the Additional file 2 (instrument subscales). There were statistically significant mean test-retest differences for some instruments, varying between 4 and 14% of the baseline score. In all measures, patients performed better in the retest than in the baseline measure. There was no considerable variance due to systematic differences over time in any assessment (σ^2^_o_ < 1) except for the 2-min walk test (σ^2^_o_ = 8.2).

**Table 3:** Test-retest reliability of measurement instruments of mobility in 65 older acute medical patients with cognitive impairment

		DEMMI	HABAM	POMA	SPPB	Gait speed ^a^ (*n* = 35)	5x chair rise test (n = 16)	2-min walk test (*n* = 35)	TUG^b^ (n = 33)	BI mobility subscale
Scale range and unit		0–100 points	0–26 points	0–28 points	0–12 points	Ratio scale (meter/second)	Ratio scale (seconds)	Ratio scale (meter)	Ratio scale (seconds)	0–40 points
Mean ± SD score 1st measure		38.5 ± 23.4	13.5 ± 7.6	10.5 ± 9.4	2.8 ± 3.2	0.62 ± 0.22	17.5 ± 4.1	71.5 ± 29.0	24.0 ± 13.5	18.7 ± 13.7
Mean ± SD score 2nd measure		38.7 ± 23.9	13.7 ± 7.8	10.9 ± 9.7	3.2 ± 3.6	0.67 ± 0.25	16.3 ± 5.2	75.9 ± 28.7	23.0 ± 12.2	19.5 ± 14.1
Mean difference absolute (95% CI)		0.3 (− 0.5 to 1.1)	0.2 (− 0.1 to 0.6)	0.4 (0.0 to 0.8)	0.4 (0.2 to 0.6)	0.04 (0.00 to 0.08)	−1.3 (− 3.1 to 0.6)	4.4 (0.8 to 8.1)	− 1.0 (− 2.6 to 0,6)	0.9 (0.1 to 1.6)
Mean difference relative to score of 1st measure		< 1%	1%	4%	14%	6%	7%	6%	4%	5%
*P* value for mean difference		0.52	0.22	0.04	< 0.001	0.04	0.16	0.02	0.22	0.03
σ^2^_p_		554.304	58.267	90.143	11.087	0.049	16.317	778.484	154.555	188.450
σ^2^_o_		0	0.01	0.062	0.074	0.001	0.44	8.221	0.169	0.288
σ^2^_residual_		5.239	1.106	1.153	0.372	0.007	5.838	55.479	9.999	4.519
ICC_AGREEMENT_		0.99	0.98	0.99	0.97	0.86	0.72	0.92	0.94	0.98
95% CI for ICC		0.99 to 0.99	0.97 to 0.99	0.98 to 0.99	0.92 to 0.98	0.75 to 0.93	0.38 to 0.89	0.84 to 0.96	0.88 to 0.97	0.96 to 0.99
P value for ICC		< 0.001	< 0.001	< 0.001	< 0.001	< 0.001	< 0.001	< 0.001	< 0.001	< 0.001
SEM_AGREEMENT_ (absolute value)		2.29	1.06	1.10	0.67	0.09	2.51	7.98	3.19	2.19
SEM_AGREEMENT_ (relative to scale range)		2.3%	4.1%	3.9%	5.6%	na	na	na	na	5.5%
SEM_AGREEMENT_ (relative to mean 1st measure)		5.9%	7.9%	10.5%	23.9%	14.5%	14.3%	11.2%	13.3%	11.7%
τ -correlation ^c^		0.30; *P* < 0.01	−0.03; *P* = 0.77	0.38; *P* < 0.01	0.66; *P* < 0.01	0.18; *P* = 0.14	0.33; *P* = 0.08	0.22; *P* = 0.08	0.43; *P* < 0.01	0.15; *P* = 0.16
Normal distribution of differences^d^		*P* < 0.001	P < 0.001	P < 0.001	P < 0.001	*P* = 0.03	P = 0.77	*P* = 0.01	P = 0.01	P < 0.001
95% LoA (log as function of X)		−0.11X + 0.3 to 0.11X + 0.3	−2.7 to 3.2	−0.22X + 0.4 to 0.22X + 0.4	−0.34X + 0.4 to 0.34X + 0.4	−0.34X + 0.04 to 0.34X + 0.04	−0.35X - 1.3 to 0.35X - 1.3	− 0.29X + 4.4 to 0.29X + 4.4	− 0.28X - 1 to 0.28X - 1	−0.26X + 0.9 to 0.26X + 0.9
MDC_90_	exact	5.3	2.5	2.6	1.5	0.21	5.8	18.5	7.4	5.1
	rounded up	6	3	3	2	na	na	na	na	6
MDC_95_	exact	6.3	2.9	3.1	1.9	0.25	6.9	22.1	8.8	6.1
	rounded up	7	3	4	2	na	na	na	na	7

*Abbreviations*: *DEMMI* De Morton mobility index, *HABAM* Hierarchical assessment of balance and mobility, *POMA* Performance oriented mobility assessment, *SPPB* Short physical performance battery, *TUG* Timed up and go test, *BI* Barthel index, *SD* Standard deviation, *CI* Confidence interval, *σ*^*2*^_*p*_ Variance between patients, *σ*^*2*^_*o*_ Variance due to systematic differences between observations, *σ*^*2*^_*residual*_ Residual variance, *ICC* Intraclass correlation coefficient, *SEM* Standard error of measurement, *LoA* Absolute limites of agreement with 95% confidence, *X* Test score, *MDC*_*90*_ Minimal detectable change with 90% confidence, *MDC*_*95*_ Minimal detectable change with 95% confidence, *na* Not applicable

^a^gait speed final score: maximum of 2 trials

^b^TUG final score: mean of 2nd and 3rd trial

^c^Kendall’s Tau correlation between absolute difference and mean scores of two measures

^d^Shapirow Wilk test of Normality

The ICC_AGREEMENT_ was above 0.9 in all outcomes except for the gait speed measure (ICC = 0.86; 95% CI: 0.75–0.93) and the 5xCRT (ICC = 0.72; 95% CI: 0.38–0.89).

For the FAC, test-retest reliability was ƙ = 0.97 (95% CI: 0.94–0.99; Table 4). Kappa values for the individual Barthel Index mobility subscale items were as follows: transfer ƙ = 0.90, mobility ƙ = 0.96 and stairs ƙ = 0.87 (Additional file 3). There were no significant differences in reliability of test scores between sub-groups of men and women, indicated by overlapping 95% CI of the ICC and ƙ values, except for the Barthel Index mobility subscale and the FAC (Additional file 4).

**Table 4:** Test-retest reliability of the Functional Ambulation Categories; *n* = 65; kappa = 0.97 (95% CI: 0.94–0.99); agreement = 92%

		Second measure scores						Observed proportion of agreement (95% CI)
		0	1	2	3	4	5	
First measure scores	0	27	0	0	0	0	0	100% (85–100)
	1	0	3	0	0	0	0	100% (31–100)
	2	0	0	0	0	0	0	na
	3	0	0	0	8	0	0	100% (60–100)
	4	0	0	0	0	16	5	76% (52–91)
	5	0	0	0	0	0	6	55% (25–82)

*CI* Confidence interval, *na* Not applicable

#### Measurement error: standard error of measurement

SEM_AGREEMENT_ values for all measurement instruments and subscales are given in Table 3 and the Additional file 2, respectively. The SEM relative to the scale range was between 2.3% (DEMMI) and 5.6% (SPPB). The SEM relative to the mean value of the first measure was between 5.9% (DEMMI) and 23.9% (SPPB).

Agreement in FAC scores between two measures was 92% (Table 4). Agreement of the Barthel Index mobility subscale items was between 58 and 62% (Additional file 3).

#### Measurement error: limits of agreement/Bland and Altman plot

The Bland and Altman plots of all measurement instruments are presented in Additional file 5. The 95% absolute limits of agreement for each instrument are listed in Table 3 and Additional file 2.

#### Measurement error: minimal detectable change

MDC_90_ and MDC_95_ values are given in Table 3 and Additional file 2.

## Discussion

The results indicate sufficient test-retest reliability for group-comparisons of the DEMMI, HABAM, POMA, SPPB, 2-min walk test, TUG, Barthel Index mobility subscale and FAC in older acute medical patients with cognitive impairment. Short-distance gait speed and chair rise measures seem insufficient (ICC < 0.9) for monitoring individual changes over time. The clinical utility of the short- and long-distance walk tests, the TUG and the chair rise test seems further limited due to significant floor effects.

### Relative test-retest reliability

The COSMIN group proposed ICC and ƙ values ≥0.7 as indicators of acceptable reliability [44]. An ICC ≥0.7 is deemed sufficient for group comparison, but for individual-level monitoring, the ICC should exceed 0.90 [45]. The ICC_AGREEMENT_ was ≥0.90 in all instruments except for the gait speed (0.86) and chair rise (0.72) measures. Thus, all instruments seem to have sufficient test-retest reliability (in terms of the consistency of within-group position), and all but the two measures mentioned seem to be suitable for the individual-level assessment of mobility over time in older acute medical patients with cognitive impairment. The results also indicate that multi-component instruments have better test-retest reliability than single-component measures.

The comparison of reliability approximations found in the present study with existing evidence is limited due to the small number of reliability studies performed with older adults with dementia and other cognitive impairment. However, there is some evidence of the test-retest reliability of physical performance measures in older (acute medical) patients with dementia, which can serve as a reference [11–13, 15–18, 20–22]. In general, and in agreement with the present study, these studies indicate sufficient test-retest reliability for most instruments, but the measurement error seems to be large and to limit the monitoring of clinically relevant intra-individual changes [17, 18, 20]. In the following, we will discuss the relative test-retest reliability of each measurement instrument under study.

### Multi-component measures of mobility

The test-retest reliability of the DEMMI and HABAM has not been examined in a well-defined group of older people with cognitive impairment before. The test-retest reliability of the DEMMI (ICC = 0.99) is very high and comparable to the intra-rater reliability reported by other authors (0.86 to 0.98) [46, 57]. In addition, the HABAM ICC value of 0.98 found in the present sample is comparable to the test-retest reliability found in two samples of mixed geriatric inpatients, assessed within one (*n* = 30; ICC = 0.99 [57]) or two (*n* = 63; ICC = 0.91 [58]) hospital days.

There is also very limited evidence on the reliability of the POMA in older people with cognitive impairment. Sterke et al. [10] reported excellent test-retest reliability for POMA total and subscale scores (ICC = 0.88 to 0.97) in 11 nursing home residents with moderate to severe dementia, a result comparable to our findings (ICC = 0.97 to 0.99).

We applied the TUG with 33 participants and found high test-retest reliability (ICC = 0.92). There is conflicting evidence for the TUG, with reliability reports ranging from 0.56 [11], 0.72 [14], 0.76 [15], 0.86 [17] and 0.58 to 0.96 [16].

No reliability studies have been performed for the mobility subscale of the Barthel Index in older people with cognitive impairment or dementia. The ICC of 0.98 in the present study is comparable to the ICC values between 0.94 and 0.96 reported for the total Barthel Index in rehabilitation patients with stroke found in a systematic review [59].

There is scarce evidence on the reliability of the SPPB in older people with cognitive impairment. Fox et al. [17] reported an ICC of 0.88 for the SPPB in a small-scale pilot study with 11 older adults with dementia who lived in residential aged care facilities. The reliability estimation found in the present study (ICC = 0.97; 95% CI: 0.92–0.98) is based on a much larger sample (*n* = 65) recruited in a different setting.

### Single-component measures of mobility

For short-distance gait speed measures, some authors have reported inconsistent reliability estimations in older people with dementia, ranging from insufficient (0.57 to 0.68) [16, 17] to excellent (0.95 to 0.97) [16, 18, 19]. Reliability estimations of gait speed test scores seem to be influenced by the research protocol and the method of gait speed assessment [60, 61]. Based on our findings, gait speed can be assessed reliably for group-comparisons (ICC = 0.86) in older acute medical patients with mild to moderate cognitive impairment if gait speed is assessed according to the methods used in this study: standing start, usual/comfortable pace, 4-m distance and the shorter time of two trials. However, short-distance gait speed measures seem to be insufficiently reliable for measuring intra-individual changes over time in this population.

We applied the 2-min walk test, a shorter version of the 6-min walk test, to assess mobility and walking endurance and found acceptable test-retest reliability (ICC = 0.92) in 35 ambulatory participants. There is evidence of reliability for the 6-min walk test only for older people with dementia. Depending on the time interval between two measures, the ICC was 0.99 (test-rest: 30–60 min; *n* = 33) [18] and 0.76 to 0.90 (intra-day and 1 week apart; *n* = 33) [16].

The test-retest reliability for timed chair rise tests has been reported to be between 0.79 and 0.96 [15, 17, 20, 22]. The ICC of 0.72 (95% CI: 0.38–0.89; *n* = 16) in the present study may deviate because of the small sample size.

To the best of our knowledge, there is no evidence for the reliability of the FAC in older people with cognitive impairment. We found a very high test-retest reliability (ƙ = 0.97), which is comparable to the excellent test-retest (ƙ = 0.95) and inter-rater (ƙ = 0.91) reliability reported for patients with acute stroke [62].

### Measurement error

Measurement error can be expressed as the SEM, the limits of agreement and MDC scores. These absolute reliability scores are easy to interpret because they are expressed in the same units as the original measure. The SEM (as % value) relative to the scale range and to the mean of the first measure allow for direct comparison of the measurement error between the measurement instruments examined in this study.

The results of our study confirm previous findings of rather large measurement error in mobility measures used with older people with dementia [17, 18, 20, 63]. The DEMMI has the smallest relative SEM and the SPPB has the largest SEM.

The limits of agreement increase by at least 20% for every retest score in all instruments except the DEMMI (11%) and the HABAM, for which the limits of agreement are − 2.7 to 3.2 points, which is 11% (0.5*5.9 points/26 points*100%) of the total scale range. For the SPPB, 5xCRT and gait speed measures, the limits of agreement increase by > 30%. These large limits of agreement and MDC scores established for most scales limit the applicability in measuring change over time in older people with cognitive impairment for several reasons: First, a change in mobility needs to be very large to exceed the measurement error. Second, the measurement error may be larger than the minimal important change, including small but clinically relevant changes. For example, the MDC_90_ for gait speed is 0.21 m/s and exceeds the median minimal important change of 0.14 m/s reported in a systematic review [64]. For the SPPB, the small meaningful change and the substantial change have been reported to be 0.27 to 0.55 points and 0.99 to 1.34 points, respectively [65]. Both values are lower than the MDC_90_ of 1.5 points found in the present study. Clinicians and researchers should consider the substantial measurement error in all scales but the DEMMI and the HABAM.

Heteroscedasticity in most data indicates a larger measurement error in higher test scores. For example, the test-retest limits of agreement for a patient with a DEMMI score of 30 points (− 3.0 to 3.6) are much lower than for a patient with a score of 70 points (− 7.4 to 8.0). The MDC values and limits of agreement presented in this study can be used to decide if a change score of an individual older person with cognitive impairment is likely to be measurement error or true change.

### Strengths and limitations

This study provides a comprehensive head-to-head comparison of the test-retest reliability of a broad set of commonly used performance-based mobility measures in older people, including instrument subscales. The selection was based on psychometric evidence, clinical feasibility and awareness [12, 13, 23, 24, 26, 27, 66–68]. Our study includes the most frequently applied instruments such as TUG, SPPB and gait speed [13, 68].

A further strength of this study is the sufficiently large [48] and consecutive sample of 65 participants, which can be judged as “good” according to the COSMIN criteria [2, 48]. However, due to significant floor effects, the sample size decreased partly but was still over the minimally acceptable threshold of *n* ≥ 30 for the timed walking tests [44].

Participants were assessed within 1 day. We aimed to include only “unchanged/stable” older people according to the definition of reliability and the recommendations on reliability study methods [2, 4]. Even though we have tried to validate the participants’ statements, the reliability of asking cognitively impaired persons about the stability of their status is not known. Further, in clinical care, it is highly unlikely that mobility is assessed twice on the same day, although frequent measurements of mobility seem worthwhile [69]. A longer interval between both study measures (e.g. 24 h or 3 days) would have been more representative of the procedures currently applied in clinical practice. In that case, however, it would have been very unlikely that unchanged/stable participants would be included, since short-time intra-individual changes in physical performance are quite common in critically ill, older acute medical patients with cognitive impairment. In a study by Hatheway et al. [70], 28% of the included older hospital patients improved their mobility and balance (HABAM) within the first 48 h. In the present study, we observed statistically significant changes of 4 to 14% in mobility performance within 1 day according to some instruments, such as the SPPB and 2-min walk test. While the individual results may still be subject to participant fatigue, all statistically significant changes observed in the present study indicate improvements in mobility. Thus, fatigue does not seem to have significantly affected overall test performances. These changes may be based on altered coordination, motor control and other facets of biological variability overlapping with fatigue. Since the DEMMI was the first measure administered, it is less susceptible for participant fatigue during an assessment session. While our results indicate otherwise, it cannot be ruled out that reliability estimations of the measurement instruments applied at the end of each session have been affected more strongly by fatigue than the DEMMI.

A further limitation of this study is that we cannot formally explain cognitive impairment based on a medical diagnosis in all participants. A diagnosis of dementia was not documented for 63% of the participants. Since cognitive impairment may be based on other pathologies or on fluctuating acute changes in mental status, such as stroke or delirium, this result is not surprising. The diagnosis of dementia can be a time-consuming process that needs longitudinal observation of the course and features of cognitive decline. Usually, it needs to be supported by reports of relatives/caregivers. This may be difficult in busy acute hospitals, where most patients stay for a short time only.

Dementia and delirium are frequently unrecognised and unreported even when present, and many clinicians find it hard to distinguish between the two disorders, especially since a great deal of overlap exists between these syndromes [71, 72]. In the present study, further misclassification may be based on participants with depression, but intact cognition, who scored low on the MMSE [72]. Another bias may result from the time span between the cognitive assessment and the study assessment of 2.6 ± 1.2 days. Since cognitive function may be fluctuating in this acute population, especially in patients with delirium, the level of cognitive function might have changed within this period of time. A more detailed and instant psychiatric review of study participants would have helped to better select and describe the study sample.

Test results of performance-based measures in older people with cognitive impairment can be influenced by the patient’s adequate motivation and attention during testing, among others [9]. These conditions usually depend on the handling, communication and experience of the assessor. The external validity of these reliability estimations might be limited by the fact that the tests were performed by a trained assessor with a quite high level of work and instrument routine. However, the test-retest reliability of other assessors should be comparable if the same strict learning procedure is followed and if the instrument is applied by the same rater at both occasions. All measures are well established and commonly used by clinicians working with older people. Furthermore, data were collected in a single hospital by one single rater only. The rater was not blinded towards the participants’ levels of cognitive impairment and the test scores of the first assessment session, which may be a major limitation of the study.

## Conclusions and implications for practice

All examined instruments show sufficient relative test-retest reliability for group comparison. Hence, these tests seem suitable for cross-sectional and interventional studies of older acute medical patients with mild to moderate cognitive impairment. For individual-level monitoring of change over time, the test-retest reliability of the short-distance gait speed and chair rise measures is insufficient (ICC < 0.9) for this purpose in this population. The clinical application of gait speed and chair rise tests should be critically considered in older acute medical patients, since ambulation and sit-to-stand transfers are applicable to a limited number of higher-functioning patients only. This limitation was also observed in the TUG and the 2-min walk test.

For the DEMMI, HABAM, POMA, TUG, SPPB, FAC, 2-min walk test and the mobility subscale of the Barthel Index, the relative reliability seems sufficient for longitudinal individual-level assessment of mobility in older people with cognitive impairment. However, MDC values and absolute reliability estimations indicate rather large measurement error for many of these instruments. This may seriously limit the detection of clinically meaningful changes over time. Clinicians and researchers should consider the substantial measurement error in most scales. The DEMMI (11%) and the HABAM (11%) were the only instruments with a measurement error (95% limits of agreement) below 20%.

## Additional files

Additional file 1: Detailed description of the assessment procedures and all measurement instruments and their subscales. (PDF 395 kb)

Additional file 2: Test-retest reliability of subscales of measurement instruments of mobility in 65 older acute medical patients with cognitive impairment. (PDF 95.6 kb)

Additional file 3: Agreement of the Barthel Index mobility subscale items. (PDF 352 kb)

Additional file 4: Test-retest reliability of measurement instruments of mobility by gender. (PDF 113 kb)

Additional file 5: Bland and Altman plots of measurement instruments of mobility, including the corresponding subscales (Figures A-N). (PDF 309 kb)

## Abbreviations

5xCRT: 5-times chair rise test
CI: Confidence interval
COSMIN: COnsensus-based Standards for the selection of health Measurement Instruments
DEMMI: De Morton Mobility Index
FAC: Functional Ambulation Categories
HABAM: Hierarchical Assessment of Balance and Mobility
ICC: Intraclass correlation coefficient
ICF: International Classification of Functioning, Disability and Health
MDC: Minimal detectable change
MMSE: Mini-Mental State Examination
POMA: Performance Oriented Mobility Assessment
SEM: Standard error of measurement
SPPB: Short Physical Performance Battery
TUG: Timed up and go test
