# Supplementary material for: Reliability of mobility measures in older medical patients with cognitive impairment
Source: BMC Geriatr. 2019 Jan 23;19:20. doi: 10.1186/s12877-019-1036-z (PMC6343264; doi:10.1186/s12877-019-1036-z)
Supplement: Supplementary file 2 — Test-retest reliability of subscales of measurement instruments of mobility in 65 older acute medical patients with cognitive impairment. (PDF 95.6 kb) [file 12877_2019_1036_MOESM2_ESM.pdf]

Additional File 2: Test-retest reliability of subscales of measurement instruments of mobility in 65 older acute medical patients with cognitive impairment

		HABAM balance	HABAM transfer	HABAM mobility	POMA balance	POMA gait
Scale range and unit		0 – 21 points	0 - 18 points	0 – 26 points	0 - 16 points	0 - 12 points
Mean ± SD score 1st measure		10.7 ± 7.4	12.1 ± 6.2	11.4 ± 8.9	6.9 ± 53.9	3.6 ± 3.8
Mean ± SD score 2nd measure		11.7 ± 7.6	12.1 ± 6.5	11.5 ± 9.1	7.3 ± 6.1	3.6 ± 3.9
Mean difference absolute (95% CI)		1.0 (-0.3 to 1.7)	0 (-0.4 to 0.4)	0.1 (-0.3 to 0.4)	0.3 (0.0 to 0.7)	0.1 (-0.1 to 0.2)
Mean difference relative to score of 1st measure		9%	0%	<1%	4%	3%
P value for mean difference		<0.01	0.93	0.70	0.06	0.50
σ^2_p		52.411	39.655	80.419	34.601	14.529
σ^2_o		0.45	0	0	0.041	0
$\sigma^2_{residual}$		4.242	1.07	0.826	1.036	0.264
$ICC_{AGREEMENT}$		0.92	0.97	0.99	0.97	0.98
95% CI for ICC		0.86 to 0.95	0.96 to 0.98	0.98 to 0.99	0.95 to 0.98	0.97 to 0.99
P value for ICC		<0.001	<0.001	<0.001	<0.001	<0.001
$SEM_{AGREEMENT}$ (absolute value)		2.17	1.03	0.91	1.04	0.51
$SEM_{AGREEMENT}$ (relative to scale range)		10.3%	5.7%	3.5%	6.5%	4.3%
$SEM_{AGREEMENT}$ (relative to mean 1st measure)		20.3%	8.5%	8.0%	15.1%	14.2%
τ -correlation [a]		-0.02; P=0.83	-0.09; P=0.41	0.18; P=0.09	0.34; P<0.01	0.47; P<0.01
Normal distribution of differences [b]		P<0.001	P<0.001	P<0.001	P<0.001	P<0.001
95% LoA (log as function of X)		-4.7 to 6.7	-2.9 to 2.9	-0.18X + 0.1 to 0.18X + 0.1	-0.34X + 0.3 to 0.34X + 0.3	-0.24X + 0.1 to 0.24X + 0.1
MDC_{90}	exact	5.0	2.4	2.1	2.4	1.2
	rounded up	5	3	3	3	2
MDC_{95}	exact	6.0	2.9	2.5	2.9	1.4
	rounded up	6	3	3	3	2

Abbreviations: HABAM = Hierarchical Assessment of Balance and Mobility; POMA = Performance Oriented Mobility Assessment; SD = standard deviation; CI = confidence interval; σ^2_p = variance between patients; σ^2_o = variance due to systematic differences between observations; $\sigma^2_{residual}$ = residual variance; ICC = intraclass correlation coefficient ; SEM = standard error of measurement; LoA = absolute limites of agreement with 95% confidence; X = test score; MDC_{90} = minimal detectable change with 90% confidence; MDC_{95} = minimal detectable change with 95% confidence

[a] Kendall’s Tau correlation between absolute difference and mean scores of two measures; [b] Shapirow Wilk test of Normality
