# Supplementary material for: Reliability of mobility measures in older medical patients with cognitive impairment
Source: BMC Geriatr. 2019 Jan 23;19:20. doi: 10.1186/s12877-019-1036-z (PMC6343264; doi:10.1186/s12877-019-1036-z)
Supplement: Supplementary file 3 — Agreement of the Barthel Index mobility subscale items. (PDF 352 kb) [file 12877_2019_1036_MOESM3_ESM.pdf]

Additional File 3: Agreement of the Barthel Index mobility subscale items

		Second measure (n)				Observed proportion of agreement (95% CI)
		**0**	**5**	**10**	**15**	
**TRANSFER item; kappa = 0.90 (95% CI: 0.83–0.97); agreement = 58%**						
**First measure (n)**	**0**	6	0	0	0	100% (52–100)
	**5**	0	16	0	0	100% (76–100)
	**10**	0	0	8	5	53% (27–78)
	**15**	0	0	2	28	80% (63–91)
**GAIT item; kappa = 0.96 (95% CI: 0.92–1.00); agreement = 62%**						
**First measure (n)**	**0**	26	0	0	0	100% (84–100)
	**5**	0	11	2	0	85% (54–97)
	**10**	0	0	16	0	84% (60–96)
	**15**	0	0	1	9	90% (54–99)
**STAIRS item; kappa = 0.87 (95% CI: 0.77–0.97); agreement = 59%**						
**First measure (n)**	**0**	39	2	1	na	93% (79–98)
	**5**	0	7	3	na	58% (27–84)
	**10**	0	0	13	na	76% (50–92)
Abbreviations: CI = confidence interval; na = not applicable						
