# Supplementary material for: Reliability of mobility measures in older medical patients with cognitive impairment
Source: BMC Geriatr. 2019 Jan 23;19:20. doi: 10.1186/s12877-019-1036-z (PMC6343264; doi:10.1186/s12877-019-1036-z)
Supplement: Supplementary file 4 — Test-retest reliability of measurement instruments of mobility by gender. (PDF 113 kb) [file 12877_2019_1036_MOESM4_ESM.pdf]

Additional file 4: Test-retest reliability of measurement instruments of mobility by gender

Measurememt instrument (continuous)	Women (n = 36, 55%)					Men (n = 29, 45%)				
	n	Mean ± SD score 1st measure	Mean ± SD score 2nd measure	$ICC_{AGREEMENT}$	95% CI for ICC	n	Mean ± SD score 1st measure	Mean ± SD score 2nd measure	$ICC_{AGREEMENT}$	95% CI for ICC
DEMMI (points)	36	43.5 ± 22.2	43.4 ± 22.0	0.99	0.99 – 0.99	29	32.2 ± 23.8	32.8 ± 15.2	0.99	0.97 – 0.99
HABAM (points)	36	14.9 ± 7.1	15.2 ± 7.5	0.98	0.95 – 0.99	29	11.7 ± 7.9	11.8 ± 7.9	0.99	0.97 – 0.99
POMA (points)	36	12.5 ± 9.3	13.1 ± 9.7	0.98	0.97 – 0.99	29	7.9 ± 9.0	8.1 ± 9.1	0.99	0.98 – 0.99
SPPB (points)	36	3.4 ± 3.2	3.8 ± 3.5	0.96	0.91 – 0.98	29	2.1 ± 3.1	2.5 ± 3.6	0.96	0.90 – 0.98
Gait speed [a] (m/sec)	23	0.61 ± 0.22	0.65 ± 0.23	0.83	0.65 – 0.92	12	0.64 ± 0.24	0.70 ± 0.29	0.93	0.72 – 0.98
5x chair rise test (sec)	11	18.3 ± 4.5	17.4 ± 5.5	0.71	0.25 – 0.91	5	15.8 ± 2.8	13.8 ± 4.0	0.67	0.00 – 0.96
2-minute walk test (m)	23	70.0 ± 27.2	73.9 ± 26.5	0.93	0.83 – 0.97	12	74.4 ± 33.3	79.8 ± 33.6	0.93	0.77 – 0.98
TUG [b] (sec)	21	23.6 ± 13.5	23.3 ± 12.4	0.94	0.86 – 0.98	12	24.7 ± 14.0	22.4 ± 11.7	0.94	0.79 – 0.98
BI mobility subscale (points)	36	21.4 ± 13.3	22.6 ± 13.6	0.96	0.91 – 0.98	29	15.3 ± 13.8	15.7 ± 13.9	1.00	0.99 – 1.00
**Measurememt instrument (categorial)**	**n**	**Median (IQR) score 1st measure**	**Median (IQR) score 2st measure**	**Kappa (K)**	**95% CI for K**	**n**	**Median (IQR) score 1st measure**	**Median (IQR) score 2st measure**	**Kappa (K)**	**95% CI for K**
FAC	36	3.5 (0 – 4)	3.5 (0 – 4)	0.93	0.88 – 0.99	29	0 (0 – 4)	0 (0 – 4)	1.00	n.c.

Abbreviations: DEMMI = de Morton Mobility Index; HABAM = Hierarchical Assessment of Balance and Mobility; POMA = Performance Oriented Mobility Assessment; SPPB = Short Physical Performance Battery; TUG = timed up and go test; BI = Barthel Index; FAC = Functional Ambulation Categories; SD = standard deviation; CI = confidence interval; ICC = intraclass correlation coefficient; IQR = interquartile range; n.c. = could not be calculated due to 100% agreement in FAC categories

[a] gait speed final score: maximum of 2 trials; [b] TUG final score: mean of 2nd and 3rd trial
