# Supplementary material for: Reliability of mobility measures in older medical patients with cognitive impairment
Source: BMC Geriatr. 2019 Jan 23;19:20. doi: 10.1186/s12877-019-1036-z (PMC6343264; doi:10.1186/s12877-019-1036-z)

Additional file 5: Bland and Altman plots of measurement instruments of mobility, including the corresponding subscales (Figures A-N)

- *In each figure, the x-axis represents the mean sores of two measures made by the same assessor and the y-axis represents the difference between the two measures.*
- *The straight line represents the mean difference between both measures; dotted lines represent the 95% upper and lower limits of agreement.*
- *The bar chart on the right side illustrates the frequency of differences between the two occasions in measurement instruments producing ordinal scale results.*

Figure	Measurement instrument	Page
A	de Morton Mobility Index	2
B	Hierarchical Assessment of Balance and Mobility	3
C	Hierarchical Assessment of Balance and Mobility balance subscale	4
D	Hierarchical Assessment of Balance and Mobility transfer subscale	5
E	Hierarchical Assessment of Balance and Mobility mobility subscale	6
F	Performance Oriented Mobility Assessment	7
G	Performance Oriented Mobility Assessment balance subscale	8
H	Performance Oriented Mobility Assessment gait subscale	9
I	Short Physical Performance Battery	10
J	Gait speed	11
K	5times chair rise test	12
L	2-minute walk test	13
M	Timed up and go test	14
N	Barthel Index mobility subscale	15

Figure A: Bland and Altman plot of the de Morton Mobility Index (DEMMI; n = 65)

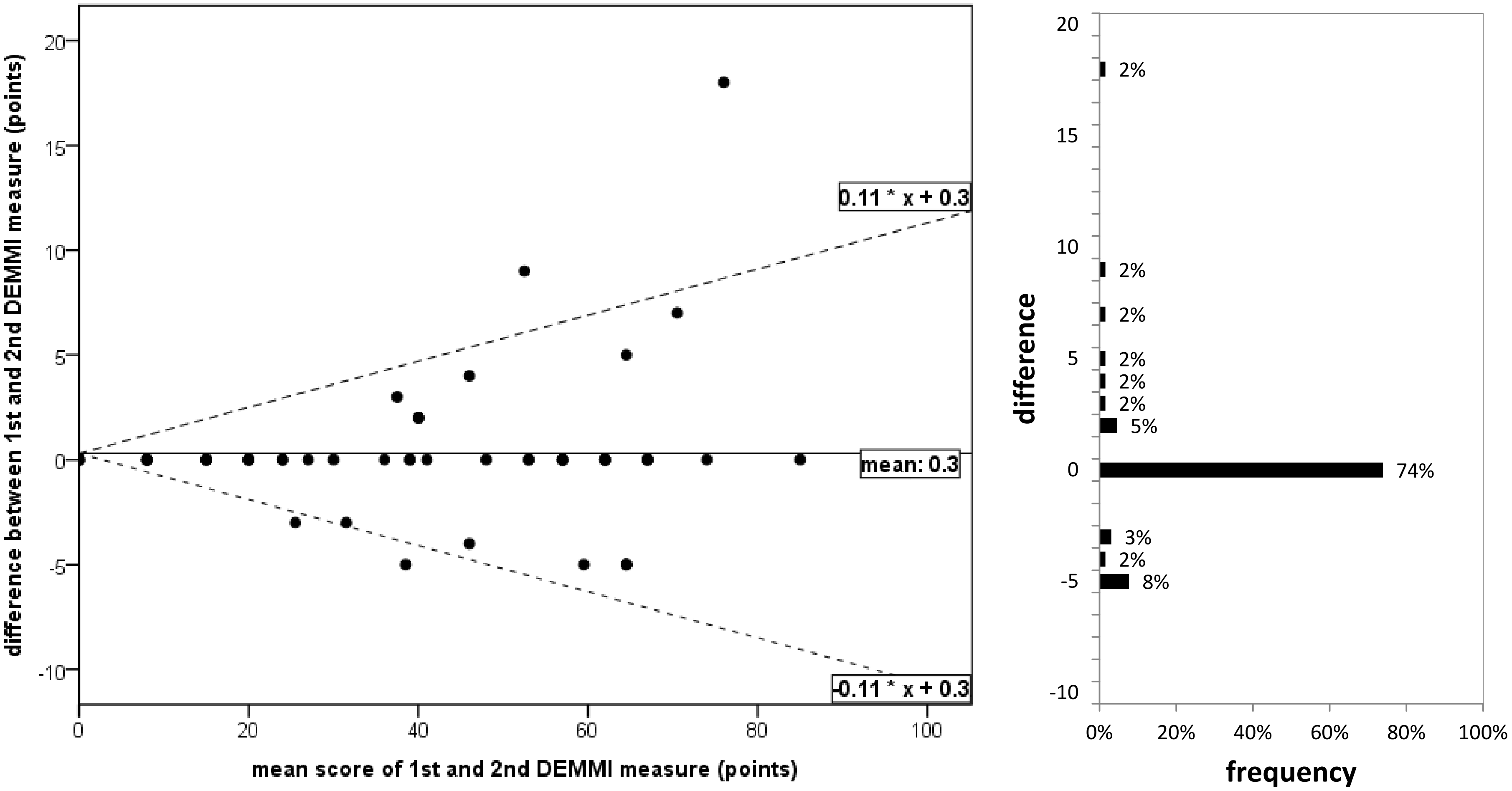

Figure B: Bland and Altman plot of the Hierarchical Assessment of Balance and Mobility (HABAM; n = 65)

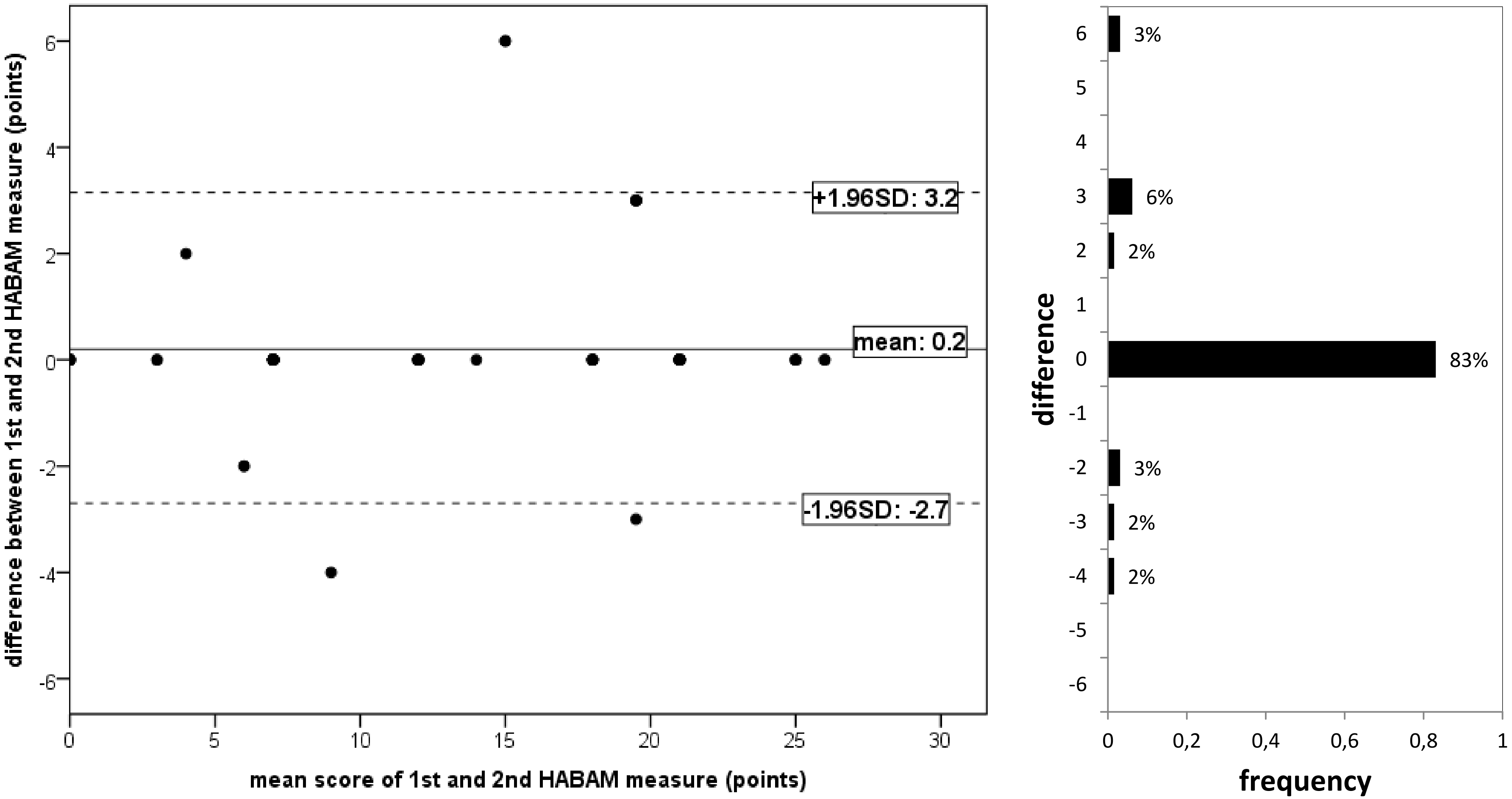

Figure C: Bland and Altman plot of the Hierarchical Assessment of Balance and Mobility (HABAM; n = 65) balance subscale

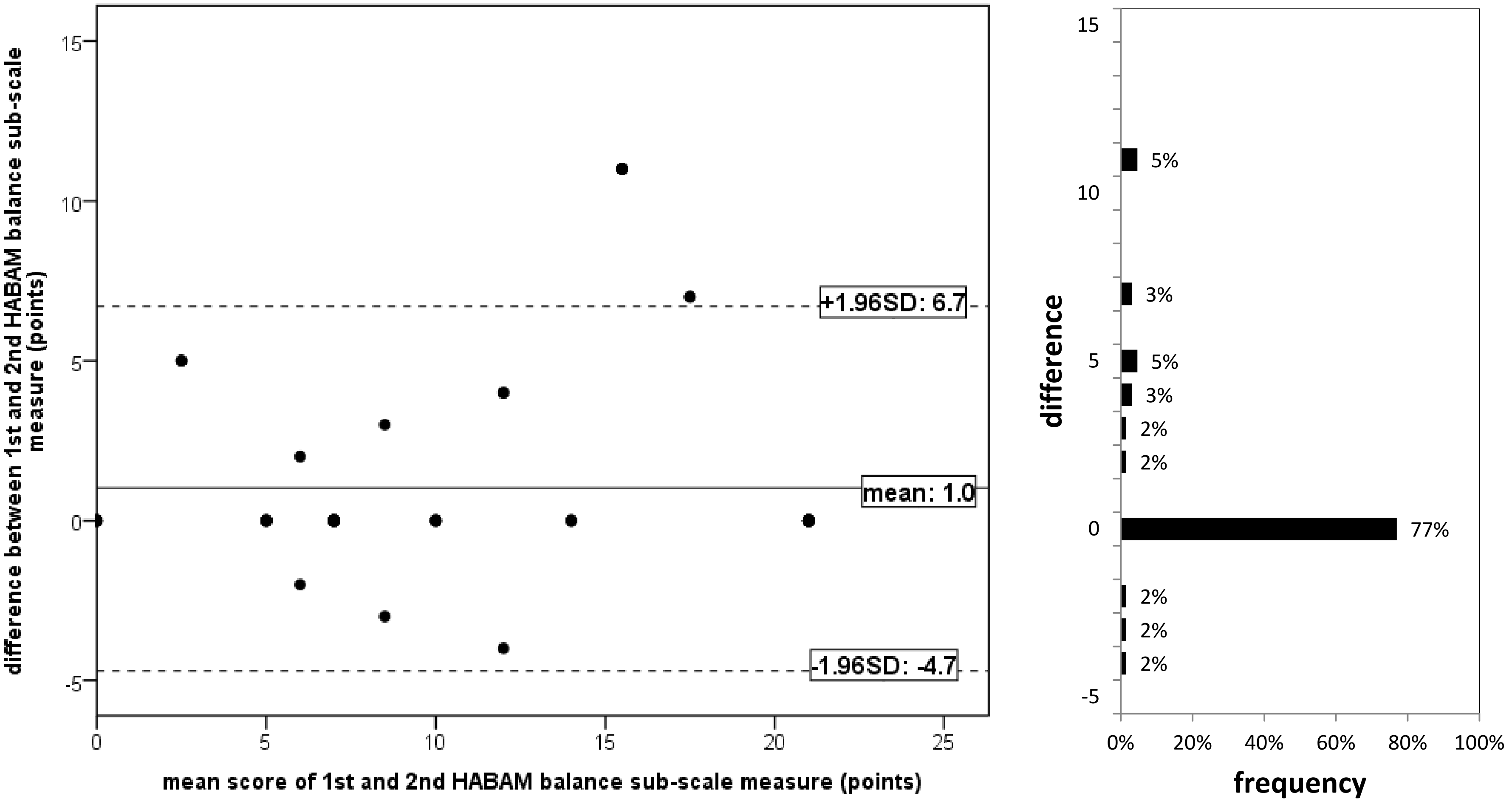

Figure D: Bland and Altman plot of the Hierarchical Assessment of Balance and Mobility (HABAM; n = 65) transfer subscale

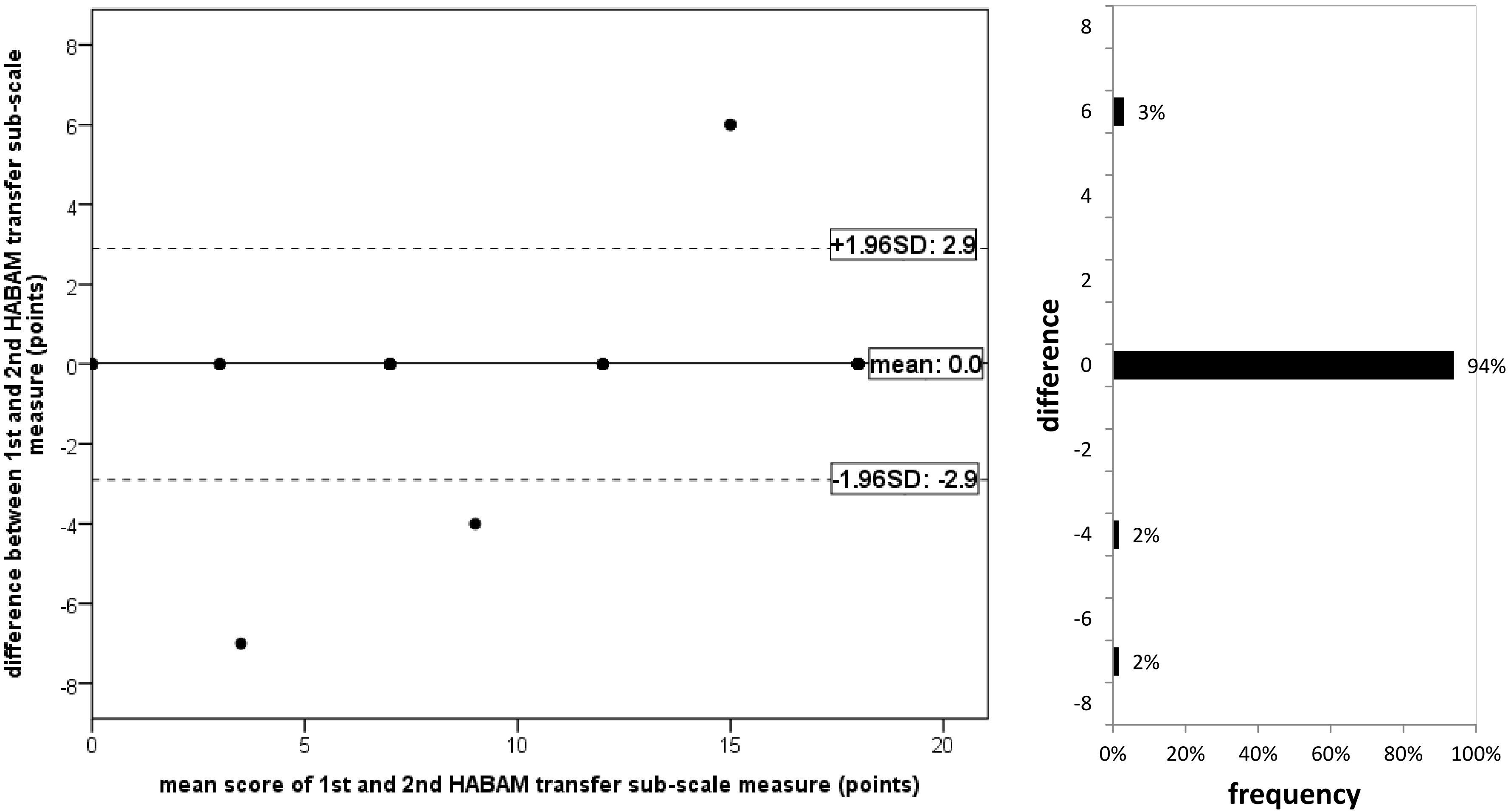

Figure E: Bland and Altman plot of the Hierarchical Assessment of Balance and Mobility (HABAM; n = 65) mobility subscale

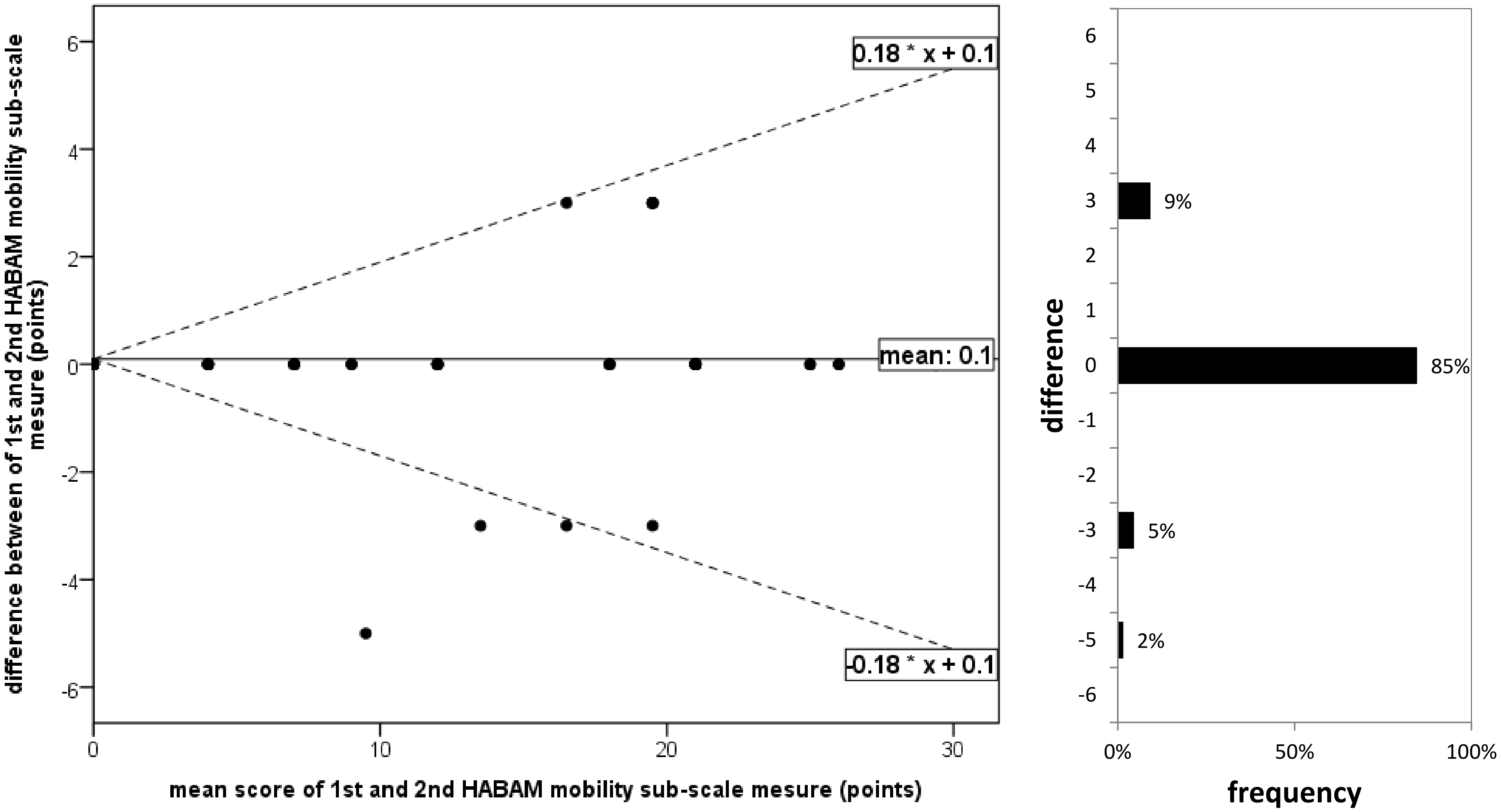

Figure F: Bland and Altman plot of the Performance Oriented Mobility Assessment (POMA; n = 65)
95% limits of agreement are based on n = 62 since for 3 participants the difference in log10 scores could not be calculated

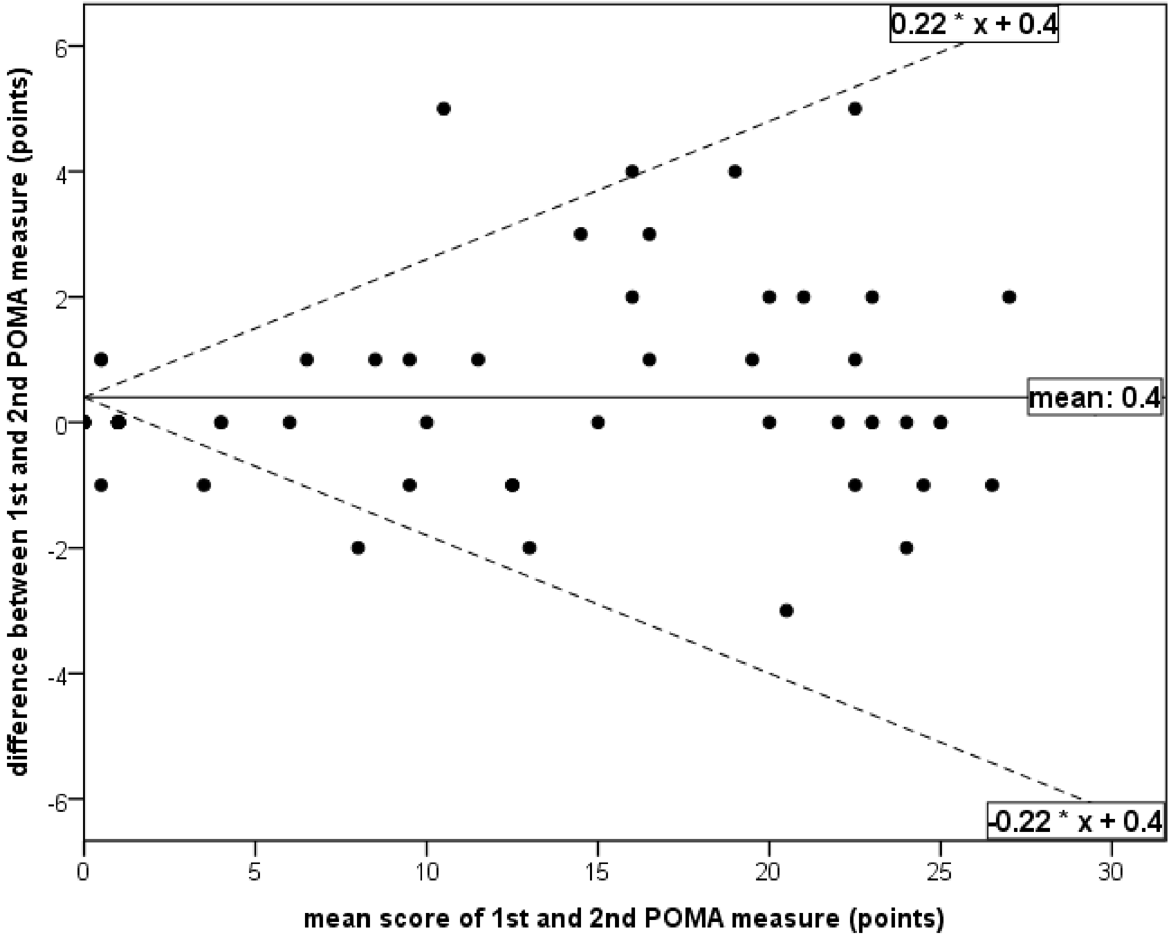

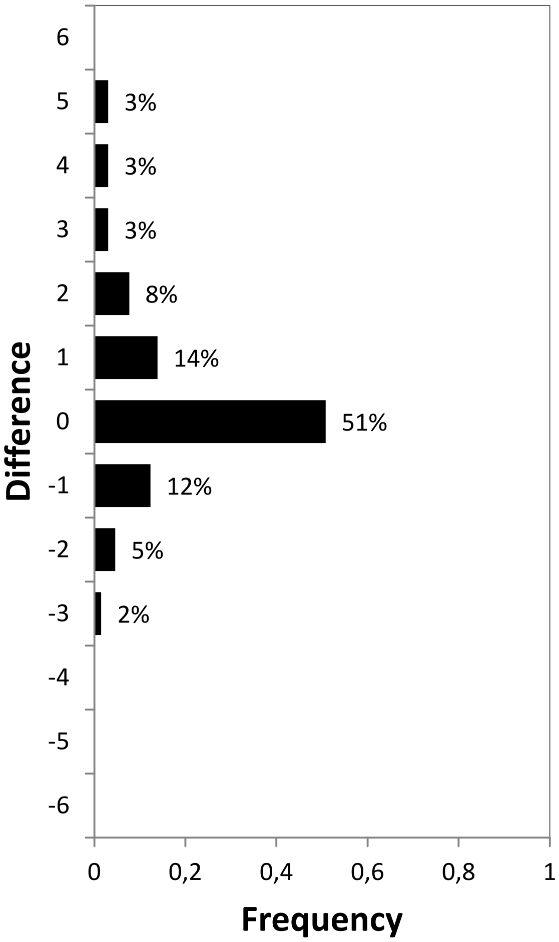

Figure G: Bland and Altman plot of the Performance Oriented Mobility Assessment (POMA; n = 65) balance subscale
95% limits of agreement are based on n = 62 since for 3 participants the difference in log10 scores could not be calculated

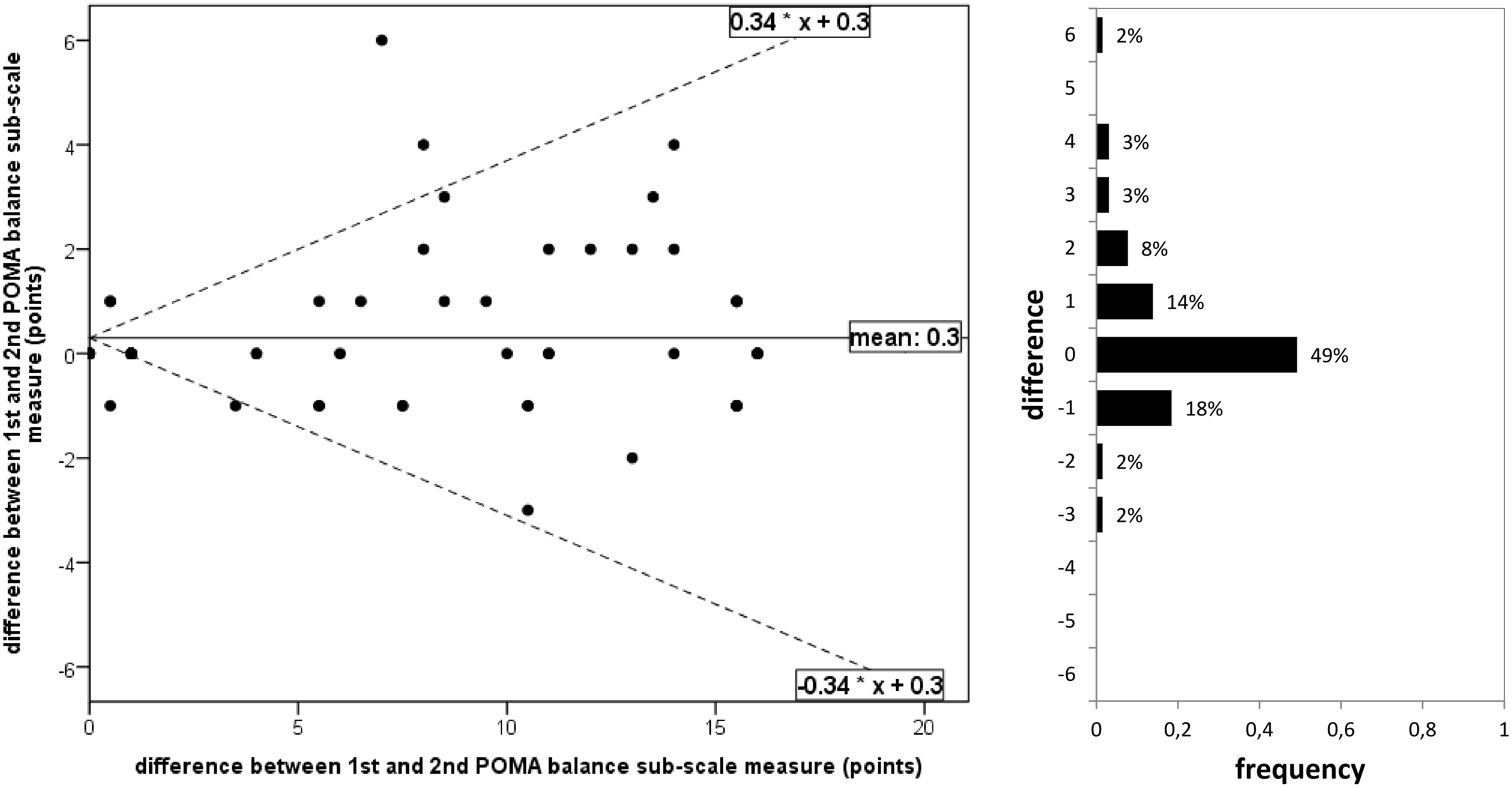

Figure H: Bland and Altman plot of the Performance Oriented Mobility Assessment (POMA; n = 65) gait subscale
95% limits of agreement are based on n = 62 since for 3 participants the difference in log10 scores could not be calculated

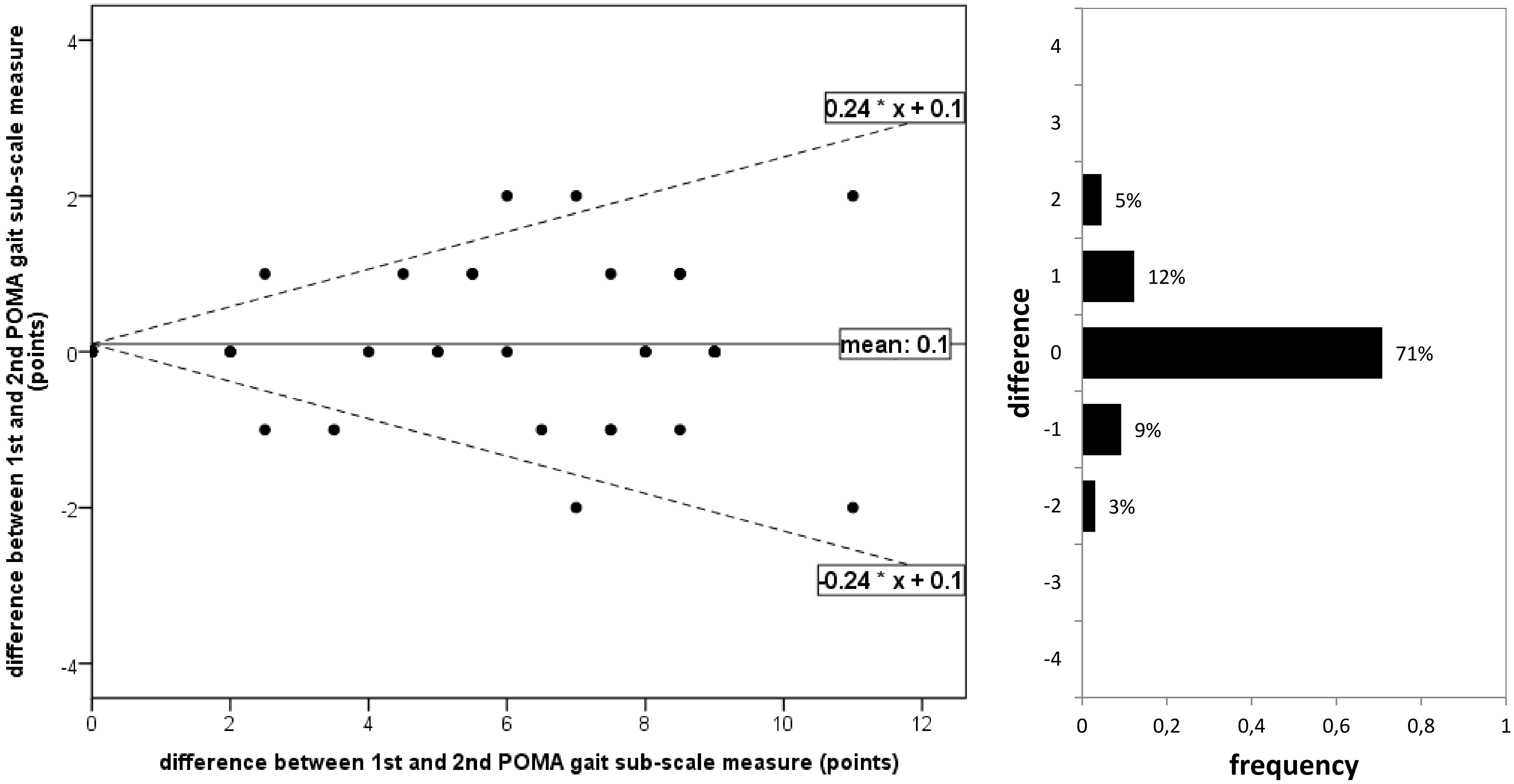

Figure I: Bland and Altman plot of the Short Physical Performance Battery (SPPB; n = 65)
95% limits of agreement are based on n = 63 since for 3 participants the difference in log10 scores could not be calculated

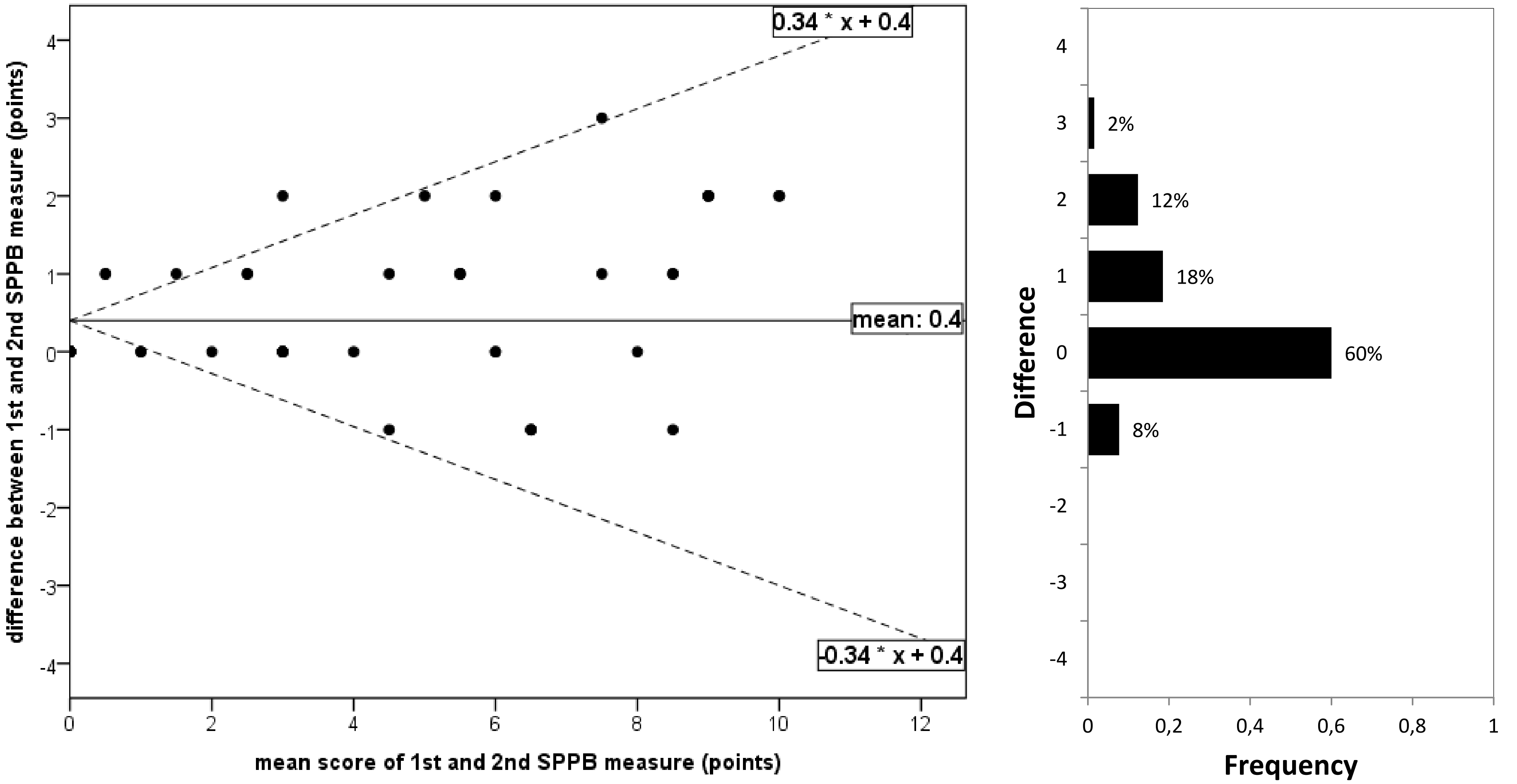

Figure J: Bland and Altman plot of the 4-meter gait speed measure (n = 35)

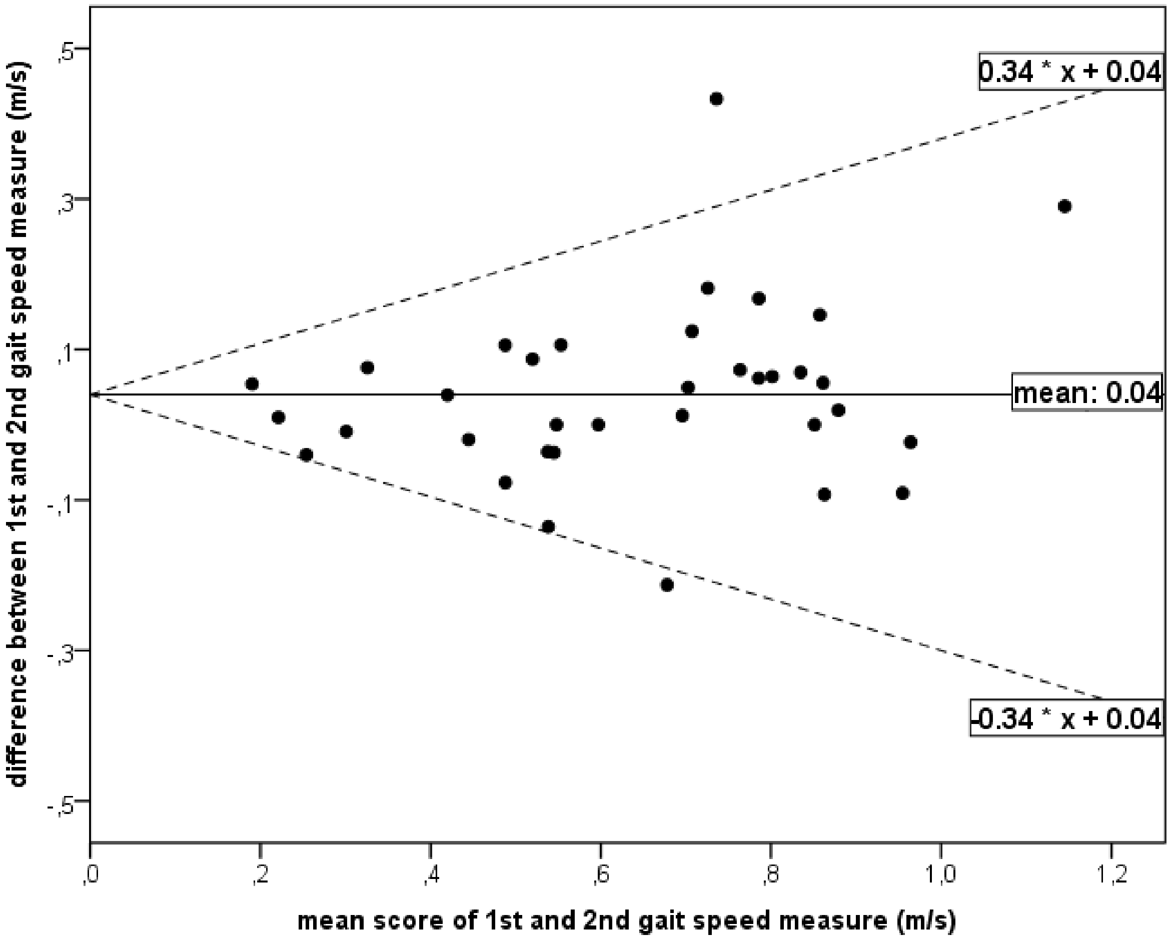

Figure K: Bland and Altman plot of the 5 times chair rise test (n = 16)

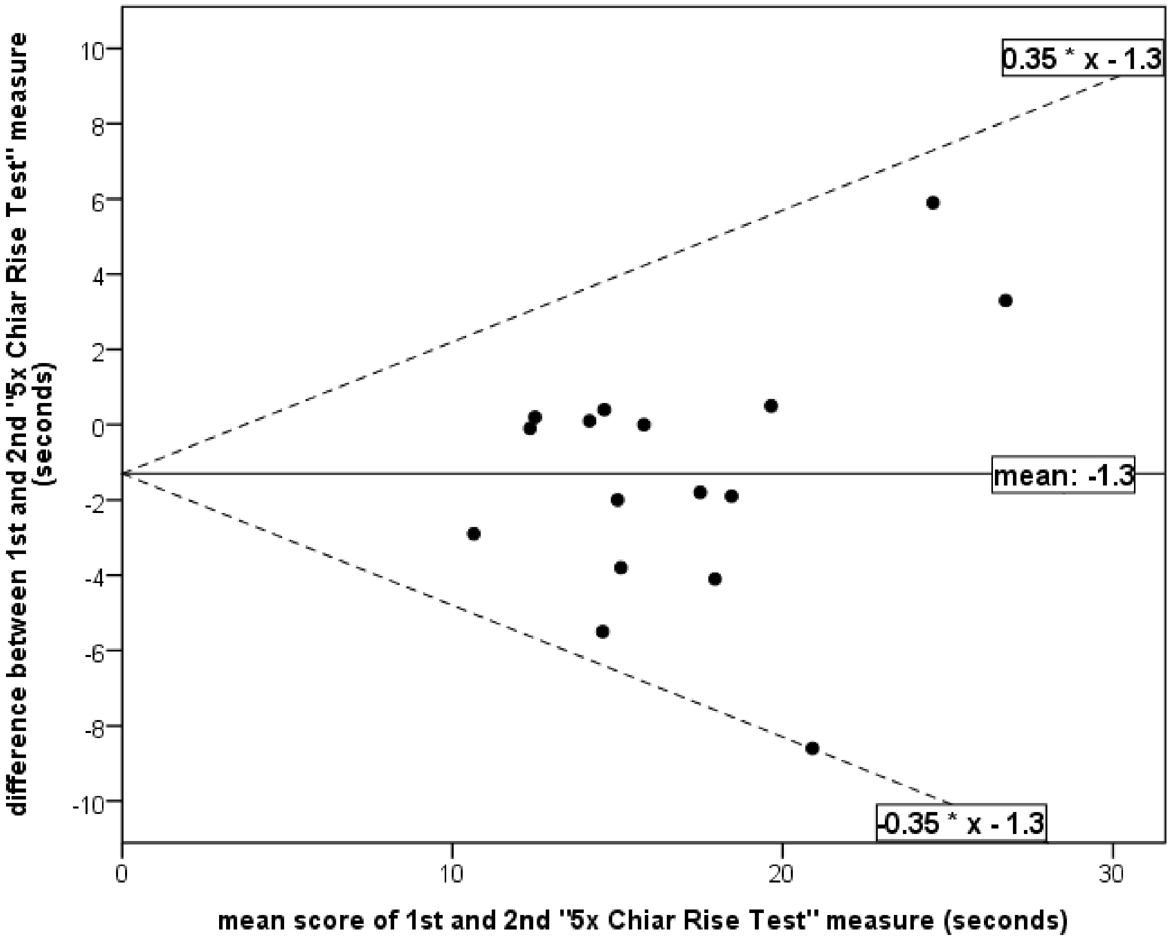

Figure L: Bland and Altman plot of the 2-minute walk test (n = 35)

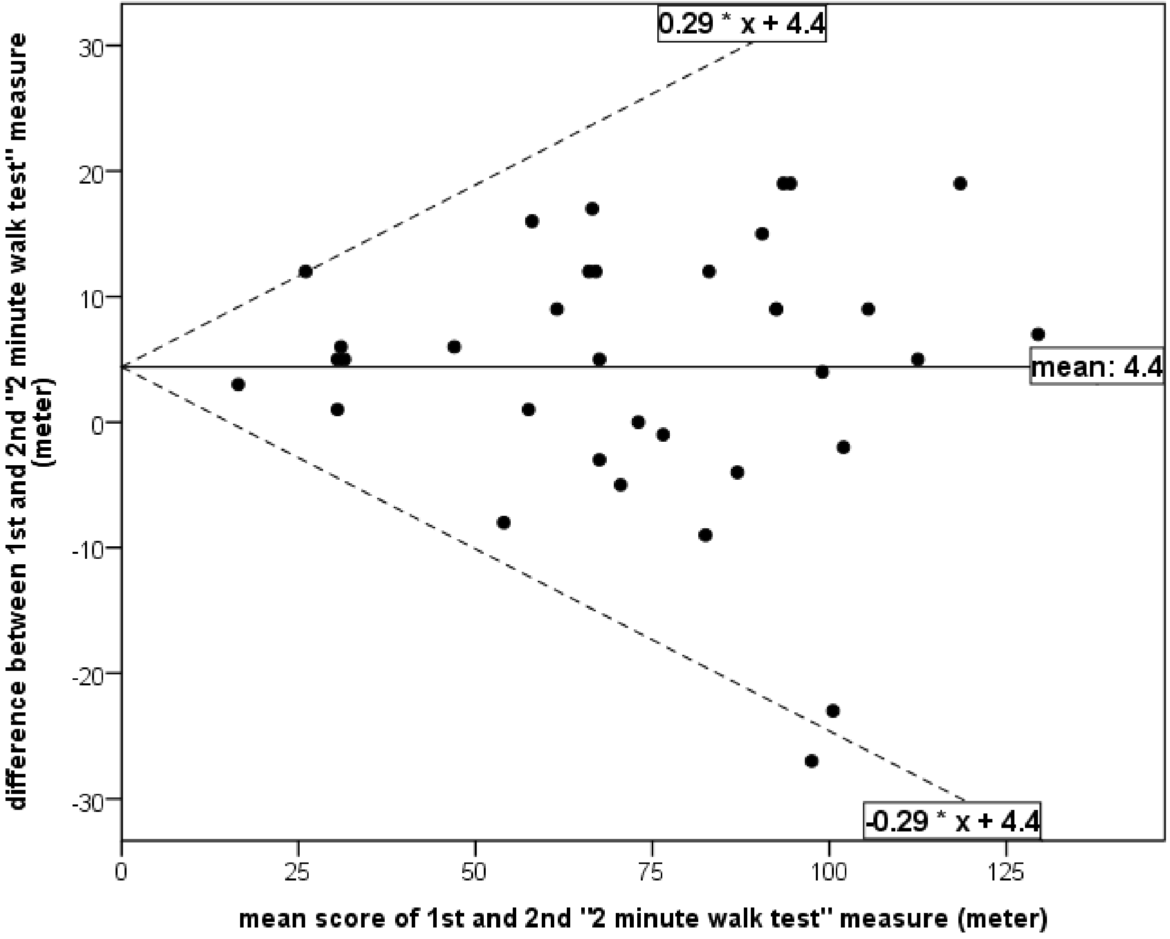

Figure M: Bland and Altman plot of the timed up and go test (TUG; n = 33)

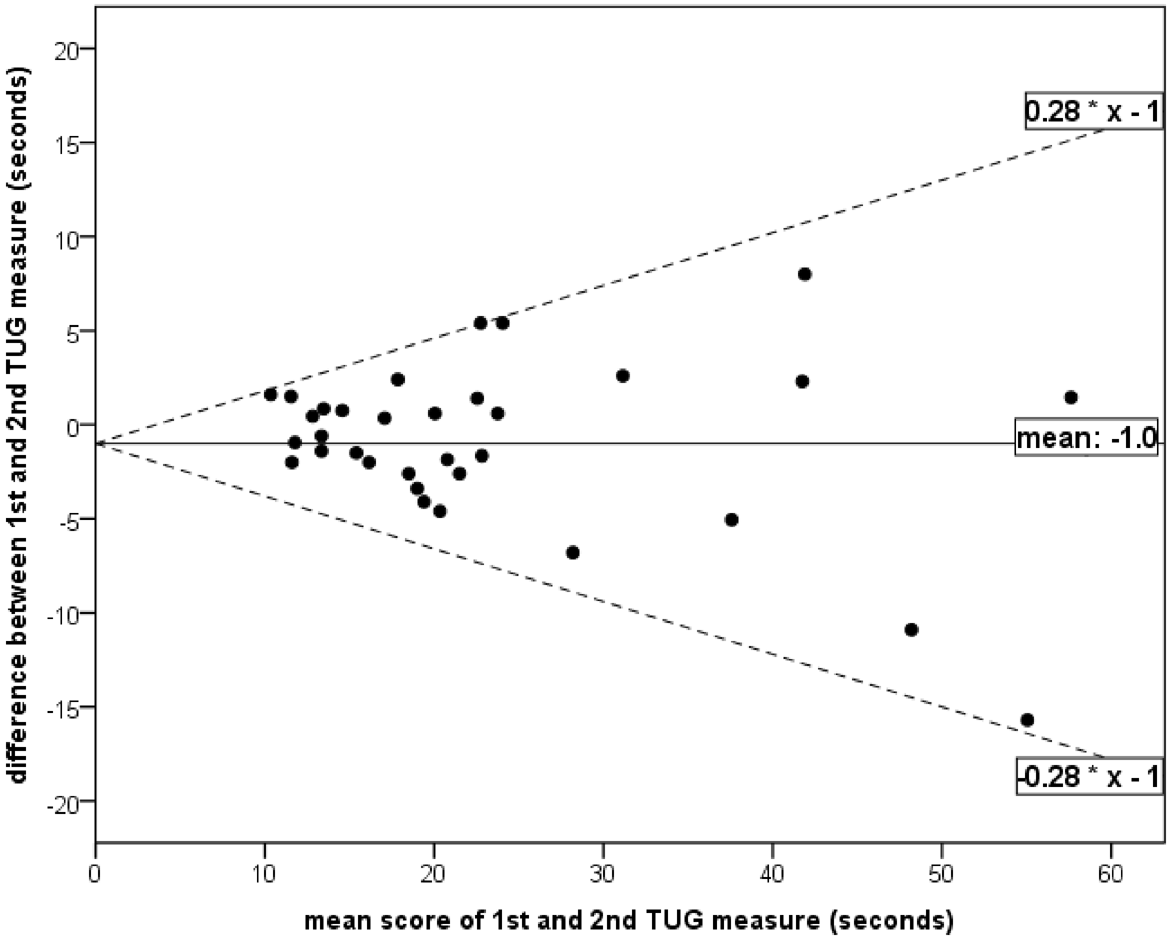

Figure N: Bland and Altman plot of the Barthel Index mobility subscale (n = 65)

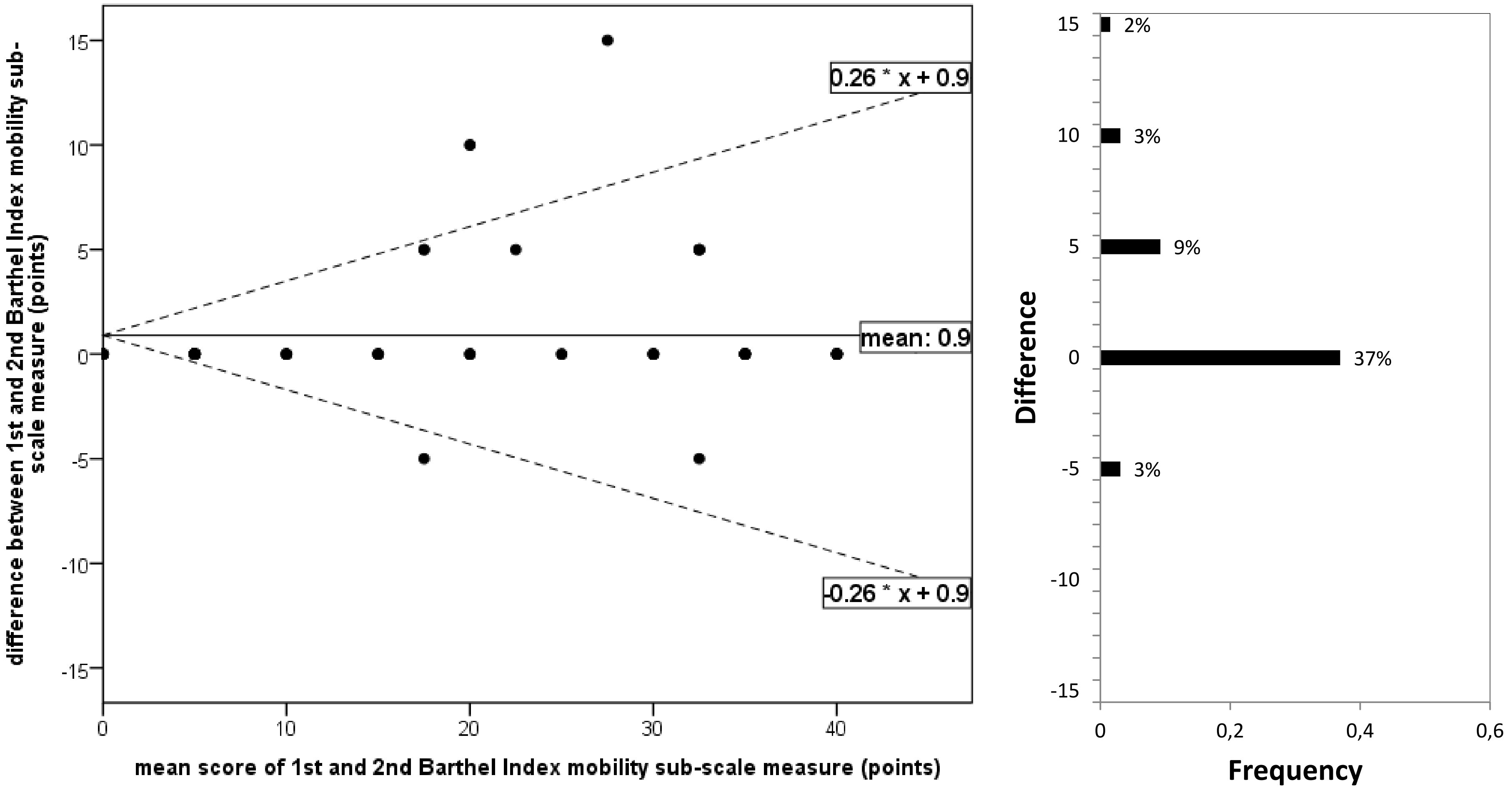

Supplement: Supplementary file 5 — Bland and Altman plots of measurement instruments of mobility, including the corresponding subscales (Figures A-N). (PDF 309 kb) [file 12877_2019_1036_MOESM5_ESM.pdf]
